# Matrine Targets BTF3 to Inhibit the Growth of Canine Mammary Tumor Cells

**DOI:** 10.3390/ijms25010540

**Published:** 2023-12-30

**Authors:** Zijian Feng, Na Sun, Fida Noor, Panpan Sun, Hua Zhang, Jia Zhong, Wei Yin, Kuohai Fan, Huizhen Yang, Zhenbiao Zhang, Yaogui Sun, Hongquan Li

**Affiliations:** 1 Shanxi Key Laboratory for Modernization of TCVM, College of Veterinary Medicine, Shanxi Agricultural University, Jinzhong 030600, China; sxaufzj@163.com (Z.F.); snzh060511@126.com (N.S.); drnoorbaloch2016@gmail.com (F.N.); sunpp0505@163.com (P.S.); zhsn060511@126.com (H.Z.); zhongjia3294@163.com (J.Z.); dkyyinwei@126.com (W.Y.); yanghz@sxau.edu.cn (H.Y.); dkyypb@163.com (Y.S.); 2 Laboratory Animal Center, Shanxi Agricultural University, Jinzhong 030600, China; fkhyxj@126.com (K.F.); zbzhangvet@sxau.edu.cn (Z.Z.)

**Keywords:** matrine, canine mammary tumors, pull-down, BTF3, ABPP

## Abstract

The canine mammary tumor model is more suitable for studying human breast cancer, and the safety concentrations of matrine and the biotin-labeled matrine probe were determined in canine primary mammary epithelial cells, and then selected canine mammary tumor cell lines CHMm and CHMp were incubated with matrine, and cell viability was detected by CCK-8. The biotin-labeled matrine probe was used to pull-down the targets of matrine in canine mammary tumor cells, and the targets were screened in combination with activity-based protein profiling (ABPP) and Genecards database, and verified by qPCR and western blot. The results showed that the maximum non-cytotoxic concentrations of matrine and biotin-labeled matrine probe in canine primary mammary epithelial cells were 250 μg/mL and 500 μg/mL, respectively. Matrine and biotin-labeled matrine probe had a proliferation inhibitory effect time-dependently on CHMm and CHMp cells within a safe concentration range, and induced autophagy in cells. Then BTF3 targets were obtained by applying ABPP and Genecards screening. Cellular thermal shift assay (CETSA) findings indicated that matrine could increase the heat stability of BTF3 protein. Pull-down employing biotin-labeled matrine probe with CHMm and CHMp cell lysates revealed that BTF3 protein was detected in the biotin-labeled matrine probe group and that BTF3 protein was significantly decreased by the addition of matrine. The qPCR and western blot findings of CHMm and CHMp cells treated with matrine revealed that matrine decreased the expression of the BTF3 gene and protein with the extension of the action time, and the impact was more substantial at the protein level, respectively.

## 1. Introduction

Breast cancer is the most frequent invasive cancer in women globally and is one of the leading causes of cancer fatalities. It also has a very diverse tumor malignancy [1,2,3]. Breast cancer overtook lung cancer in terms of new cases in 2020, taking first place in global cancer incidences, and around one in every eight women globally will acquire breast cancer [4,5]. Clinically, there are four sub-types of breast cancer; (i) Luminary A, (ii) Luminary B, (iii) HER2 over-expression, and (iv) triple-negative breast cancer [6,7]. Breast cancer treatment primarily consists of breast-conserving surgery, mastectomy with adjuvant radiotherapy and chemotherapy, and the use of targeted therapies for various types of breast cancer, such as trastuzumab, which can significantly improve the survival rate of patients with HER2-positive breast cancer [8], and the selective estrogen receptor modifier (SERM), which binds to estrogen in a competitive manner and inhibits cell proliferation and growth [9]. Although therapeutic advances have significantly improved the survival rate of breast cancer patients, the high risk of breast cancer recurrence remains a clinical concern, and poor prognosis is frequently accompanied by breast cancer metastasis, with the liver, brain, bone, and lung being among the target organs for metastasis [10,11,12,13]. Gene mutations or changes in gene expression have a role in the spread of breast cancer, and the next area of study will be understanding the mechanism behind breast cancer metastasis and investigating the targets of intervention related to this process. Tumors from different species show cross-species similarities, and in addition to anatomical and physiological similarities between dogs and humans, canine mammary tumors also exhibit major pathological features of human cancers, including long-term carcinogenesis, tumor heterogeneity, treatment resistance, and metastasis [14,15,16]. The wholesome and transcriptome analyses of 191 spontaneous canine mammary tumors and the comparison of the signaling pathways involved in human mammary tumor development revealed that there was a significant degree of overlap between the two, including the “PI3K/AKT”, “PI3KCA mutation”, and “P53 pathway” [17]. These indicate that canine mammary tumors and human mammary cancers have significant similarities in carcinogenesis, and the canine mammary tumor model is a suitable model for the study of human mammary cancer.

Matrine was initially isolated and extracted as an alkaloid from the root bark of Sophora flavescens Aiton and later, matrine was also found in Sophora alopecuroides, the roots of mountain bean, and other leguminous Sophora plants [18]. Matrine has a wide range of pharmacological effects; matrine possesses antiviral activities via inhibiting porcine reproductive and respiratory syndromevirus (PRRSV) and porcine circovirus type 2 (PCV2) replication in mice and cells [19]. Matrine also plays an important role in PAM co-stimulated with PRRSV 5′UTR RNA and LPS via its effect on NF-κB and the NLRP3 inflammasome, and matrine treatment alleviated the disease severity of ongoing experimental autoimmune encephalomyelitis (EAE) and reduced inflammatory infiltration and demyelination [20]. Matrine inhibited cell proliferation and induced apoptosis in lung cancer A549 and 95D cells in a dose- and time-dependent manner and caused Hep3B cell apoptosis [21,22], and matrine can inhibit the proliferation and metastasis of gastric cancer cells through the PI3K/Akt/uPA pathway [23]. In breast cancer research, it has been shown that matrine can significantly inhibit the growth of MCF-7 and MDA-MB-231 and induce apoptosis in these cells [24,25]. However, the direct targets of matrine involved in the development of breast cancer have not been reported yet.

The first step in drug discovery and development is the correct identification and validation of drug–target interactions, as well as chemical proteomics which can dissect the complex interactions between the drug–target. Thus far, there have been tremendous technological advances in the identification of active molecules and targets [26]. For example, small molecules, such as matrine, block or activate target protein activity by interacting directly with them, and activity-based protein profiling (ABPP) is a technique wherein that pull-down probe owning an active chemical moiety can be covalently attached to a target protein in an active site and can identify small molecule targets [27]. In this study, we applied a biotin-labeled matrine probe to a canine mammary tumor in situ and metastatic cell lines, applied ABPP to search for the common target of matrine in the two cell lines, and verified it at the molecular level, revealing the molecular mechanism of canine mammary tumor inhibition of proliferation, providing strong fundamental support for the development of matrine as a targeted cancer therapy drug.

## 2. Results

### 2.1. Maximum Safe Concentration of Matrine and Biotin-Labeled Matrine Probe

**Figure 1:**
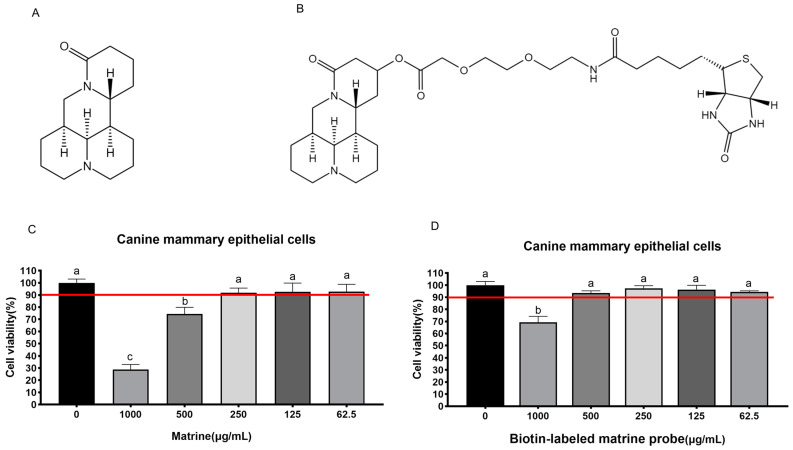
Determination of the safe concentration of matrine and biotin-labeled matrine probe in canine mammary epithelial cells. (**A**): Structural formula of matrine; (**B**): Structural formula of biotin-labeled matrine probe; (**C**): Safe concentration of matrine in canine mammary epithelial cells is 250 μg/mL; (**D**): Safe concentration of biotin-labeled matrine probe in canine mammary epithelial cells is 500 μg/mL. Significance assessed with one-way ANOVA, different letters indicate a significant difference (*p* < 0.05).

The maximum safe concentrations of matrine (Figure 1A) and biotin-labeled matrine probe (Figure 1B) were determined by CCK-8 in canine primary mammary epithelial cells, and the results showed that matrine and biotin-labeled matrine probe had toxic effects on cells at 500 μg/mL and 1000 μg/mL, however, the cell survival rate was greater than 90% below these concentrations, so it can be concluded that the maximum safe concentrations of matrine and biotin-labeled matrine probe (Figure 1C,D) in canine mammary epithelial cells are 250 μg/mL and 500 μg/mL, respectively.

### 2.2. Cell Proliferation Assays

**Figure 2:**
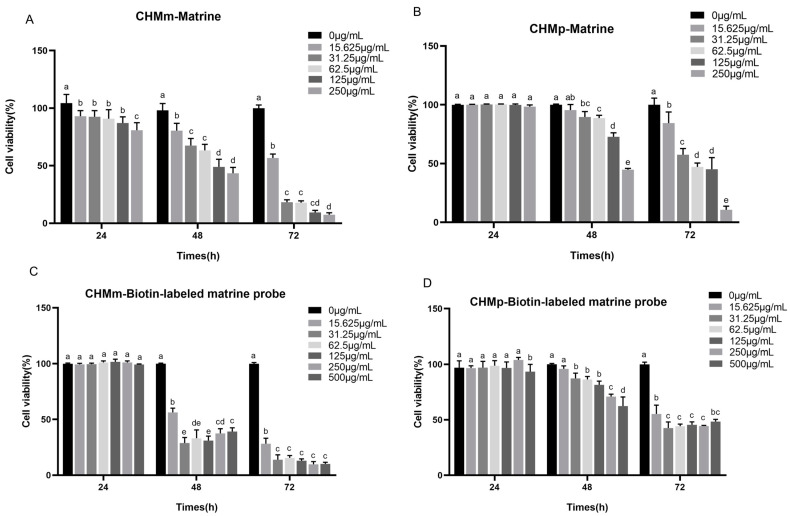
Proliferation inhibition of canine mammary tumor cell lines by matrine and biotin-labeled matrine probe. (**A**): Proliferation inhibition of CHMm cells by matrine; (**B**): Proliferation inhibition of CHMp cells by matrine; (**C**): Proliferation inhibition of CHMm cells by biotin-labeled matrine probe; (**D**): Proliferation inhibition of CHMp cells by biotin-labeled matrine probe. Significance assessed with one-way ANOVA, different letters indicate a significant difference (*p* < 0.05).

The maximal safe concentrations of matrine and biotin-labeled matrine probe in canine mammary epithelial cells were 250 μg/mL and 500 μg/mL, respectively, and these concentrations of matrine and biotin-labeled matrine probe were serially diluted two-folds and applied to the groups of CHMp and CHMm cells. Compared to the control group, the results showed that different concentrations of matrine and biotin-labeled matrine probe significantly inhibited the proliferation of CHMm and CHMp cells (Figure 2A,B), the inhibition rate was proportional to time, and the inhibitory effect of biotin-labeled matrine probe on canine mammary tumor cells was similar to that of matrine (Figure 2C,D).

### 2.3. Scanning Electron Microscope (SEM) and Transmission Electron Microscope (TEM) Analysis

**Figure 3:**
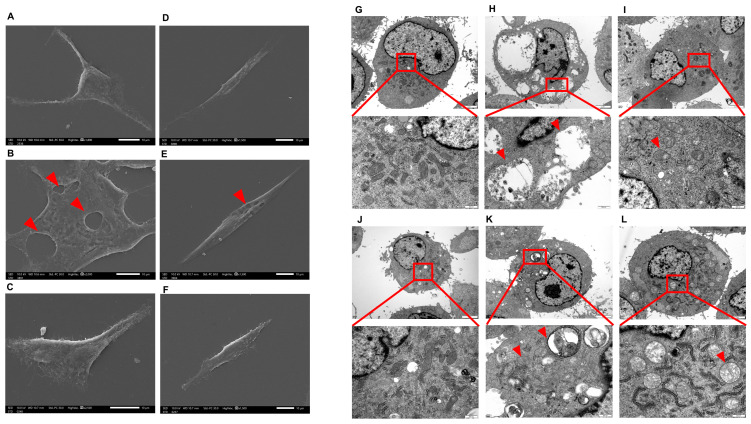
SEM and TEM observed the effect of matrine on canine mammary tumor cell lines (**A**): CHMm cell control group observed by SEM; (**B**): 250 μg/mL matrine action on CHMm cells observed by SEM; (**C**): 62.5 μg/mL matrine action on CHMm cells observed by SEM; (**D**): CHMp cell control group observed by SEM; (**E**): 250 μg/mL matrine action on CHMp cells observed by SEM; (**F**): 62.5 μg/mL matrine action on CHMm cells observed by SEM; (**G**): CHMm cell control group observed by TEM; (**H**): 250 μg/mL matrine action on CHMm cells observed by TEM; (**I**): 62.5 μg/mL matrine action on CHMm cells observed by TEM; (**J**): CHMp cell control group observed by TEM; (**K**): 250 μg/mL matrine action on CHMp cells observed by TEM; (**L**): 62.5 μg/mL matrine action on CHMm cells observed by TEM.

The CHMm and CHMp cells were treated with 250 μg/mL, 62.5 μg/mL for 36 h; SEM showed that compared with the control group, vacuolated lesions appeared on the cell surface of CHMm and CHMp cells at 250 mg/mL matrine concentration, and there was no significant change on the cell surface of the 62.5 μg/mL matrine cells (Figure 3A–F, shown by the red arrow in the figure). TEM showed that compared with the control group, autophagy occurred in CHMm and CHMp at a 250 mg/mL matrine concentration, with autophagic vesicles appearing in CHMp and most of CHMm in the late stage of autophagy, and mitochondrial swelling occurred in both cells; autophagy was reduced in CHMm and CHMp at a dose of 62.5 mg/mL matrine concentration, but mitochondrial swelling was obvious (Figure 3G–L, shown by the red arrow in the figure).

### 2.4. Enrichment of Matrine Targets

**Figure 4:**
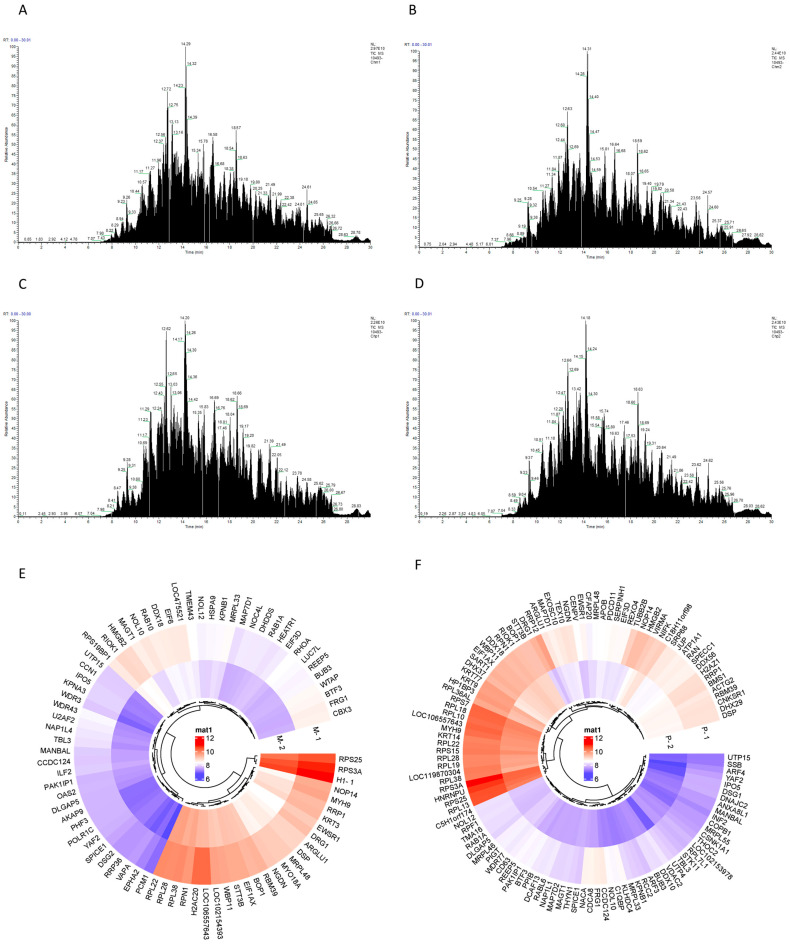
Potential targets of matrine action in canine mammary tumor cell lines. (**A**): CHMm competition group TIC total ion chromatogram; (**B**): CHMm probe group TIC total ion chromatogram; (**C**): CHMp competition group TIC total ion chromatogram (**D**): CHMp probe group TIC total ion chromatogram; (**E**): Targets of action with CHMm “Matrine Combined Score” ≥ 1.5 (M-1 for CHMm probe group, M-2 for CHMm competition groups); (**F**): Targets of action with CHMp “Matrine Combined Score” ≥ 1.5 (P-1 for CHMp probe group, P-2 for CHMp competition groups).

According to the results of LC-MS/MS high-resolution mass spectrometry, 949 and 711 targets were detected in the CHMm competition and the probe groups, respectively (Figure 4A,B), and in the CHMp competition and probe groups, 810 and 685 targets were detected, respectively (Figure 4C,D). These were quantitatively compared to the target contents based on samples of the intensity of the targets in the probe and competition groups. The expression intensity ratio of CHMm and CHMp in the probe group to the competition group was referred to as the “Matrine Combined Score”, and when the “Matrine Combined Score” was ≥1.5 it was considered a potential target of matrine for canine mammary tumor cells. Between the two larger ratios, it was indicated that the stronger the matrine competition for the combined targets, the more likely it was a potential target of matrine; thus, 79 targets were produced by the CHMm screen (Figure 4E) and 119 by the CHMp screen, respectively (Figure 4F).

### 2.5. GO and KEGG Analysis of Potential Targets of MATRINE

**Figure 5:**
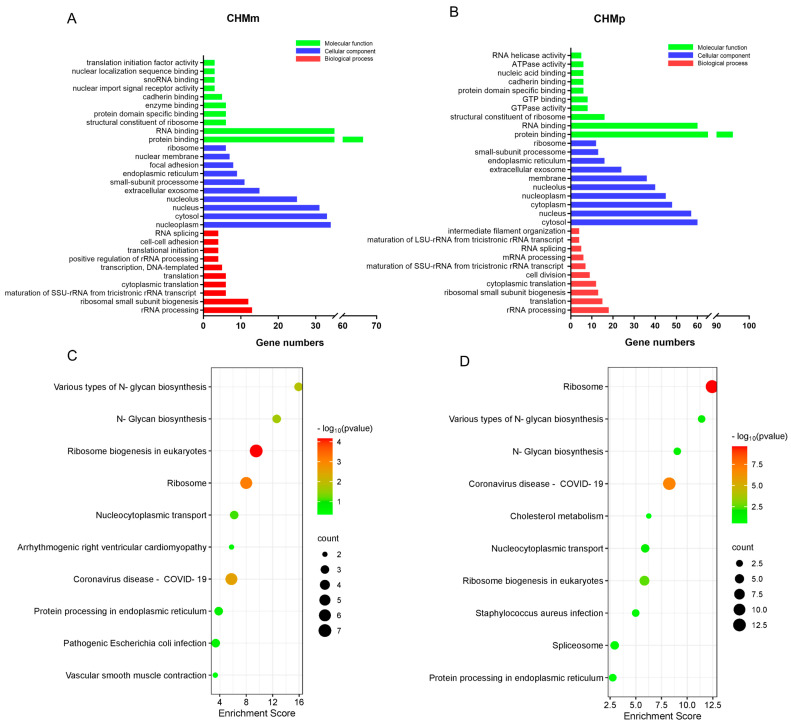
GO and KEGG analysis of potential targets of matrine in canine mammary tumor cell lines. (**A**): GO analysis of matrine potential targets of action in CHMm; (**B**): GO analysis of matrine potential targets of action in CHMp; (**C**): KEGG analysis of matrine potential targets of action in CHMm; (**D**): KEGG analysis of matrine potential targets of action in CHMp.

The above-mentioned potential target of matrine in CHMm and CHMp were imported into the DAVID database, and the results of the GO enrichment analysis and KEGG pathway analysis were obtained. According to the findings, the GO and KEGG enrichment results of matrine in CHMm and CHMp partially overlapped, and the GO analysis results showed that the biological process mainly focused on rRNA processing, cytoplasmic translation, molecular functions which focus on the nucleolus, ribosome, and cellular components mainly focused on RNA binding, as well as protein binding (Figure 5A,B), whereas KEGG analysis was mainly focused on ribosomes, various types of N-glycan biosynthesis, and other pathways (Figure 5C,D).

### 2.6. PPI Protein Interaction Network Construction

**Figure 6:**
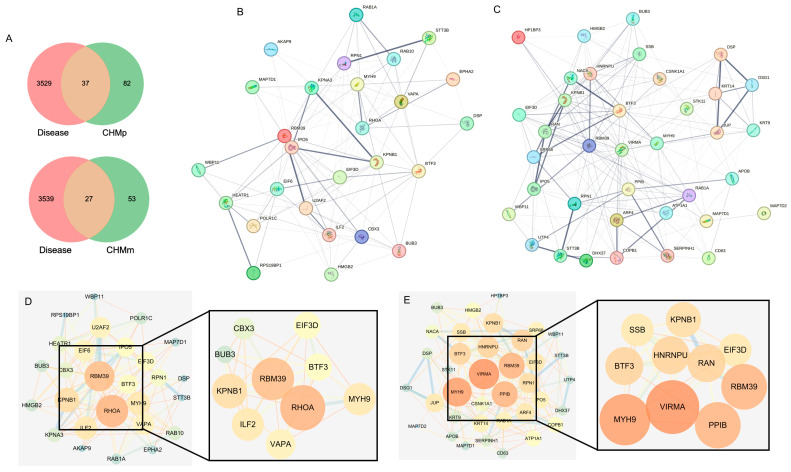
Screening of core targets for matrine anti-canine mammary tumor cell lines. (**A**): Matrine activity targets linked to “canine mammary cancers”; (**B**): Protein–protein interaction network for 27 CHMm-related targets; (**C**): Protein–protein interaction network for 37 CHMP-related targets; (**D**): Degree-based CHMm target screening; (**E**): Degree-based CHMp target screening.

The CHMm and CHMp were screened for 27 and 37 targets, respectively (Figure 6A). We accessed the STRING database, imported the targets screened for CHMm and CHMp, obtained the network interactions map between targets (Figure 6B,C), and visually analyzed them using Cytoscape3.7.2. We then compared this to the CHMm network graph having 27 nodes and 122 edges and the CHMp network graph having 37 nodes and 198 edges. Using the cytoHubba tools to screen the targets in the network interoperability graph, CHMm and CHMp obtained 10 core targets, respectively (Figure 6D,E). We then took the intersection of the core targets obtained from the two screenings and obtained five matrine anti-canine mammary tumor cell line action targets (Table 1), imported the targets into the UniProt database to obtain the full name of the genes, and ranked the targets based on the “Matrine Combined Score.”

### 2.7. BTF3 Is the Specific Binding Protein of Matrine

**Figure 7:**
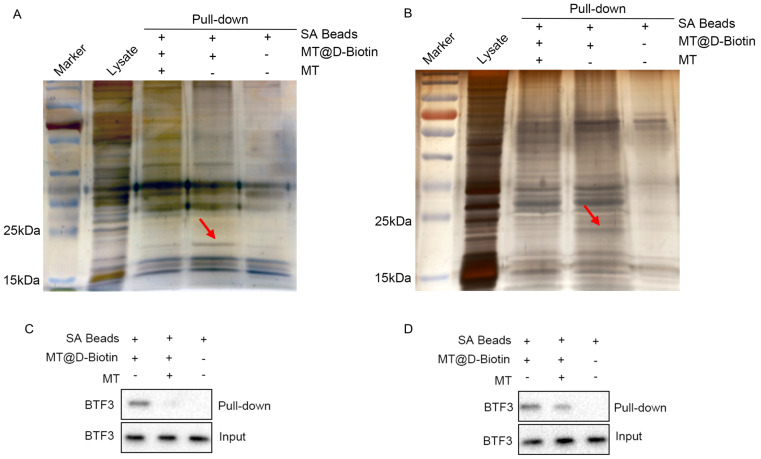
Pull−down search for matrine−specific effector proteins. (**A**): CHMp cell lysate pull−down followed by silver nitrate to search for matrine−specific binding proteins; (**B**): CHMm cell lysate pull−down followed by silver nitrate to search for matrine−specific binding proteins; (**C**): CHMp cell lysate pull−down followed by Western Blot to validate BTF3 protein expression; (**D**): CHMm cell lysate pull−down followed by Western Blot to validate BTF3 protein expression.

The results of silver nitrate staining showed that the biotin-labeled matrine probe groups of all CHMp and CHMm cell lines had specific bands between 15–25 kDa, and the bands of the biotin-labeled matrine probe + matrine group were significantly weakened (Figure 7A,B, shown by the red arrow in the figure), indicating that matrine competitively binds to this protein, and it was guessed that the matrine-specific binding protein, combined with the above screening results, targets protein molecule sizes between 15–25 kDa, which are a basic transcription factor of 3 (BTF3). Subsequent western blot validation revealed that BTF3 protein was highly expressed in the CHMm and CHMp biotin-labeled matrine probe group, whereas BTF3 protein was significantly reduced in the biotin-labeled matrine probe + matrine group, and no bands appeared in the blank magnetic bead control group (Figure 7C,D). This indicates that BTF3 is a specific binding protein for matrine in canine mammary tumor cell lines.

### 2.8. Matrine and BTF3 Molecular Docking Simulations

**Figure 8:**
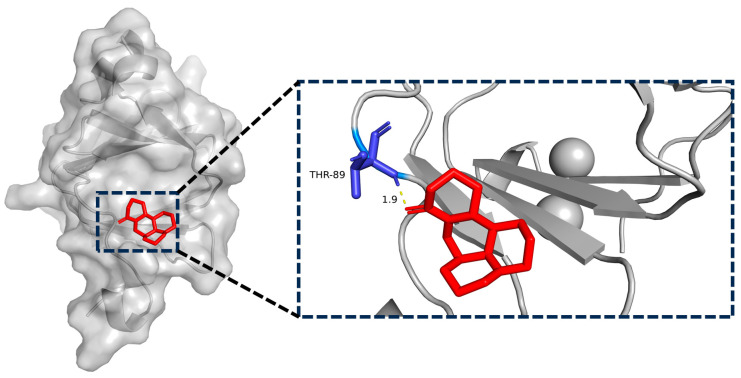
Simulation of molecular docking of matrine with BTF3 targets and their binding sites.

In recent years, researchers have focused heavily on the use of computers and structural biology in drug design in order to identify small molecule targets and their actions [28]. BTF3 protein and matrine were imported into AutoDockTools1.5.6 software for molecular docking simulation to elaborate on their molecular level. According to these data, the binding energy between matrine and BTF3 was −5.8 kcal/mol, and matrine formed a 1.9 Å length hydrogen bond with THR-89 amino acid. All of this implies that the interaction between matrine and BTF3 protein was stable, and that THR-89 amino acid was most likely the direct binding site of matrine (Figure 8).

### 2.9. CETSA Identifies BTF3 as a Target of Matrine Action

**Figure 9:**
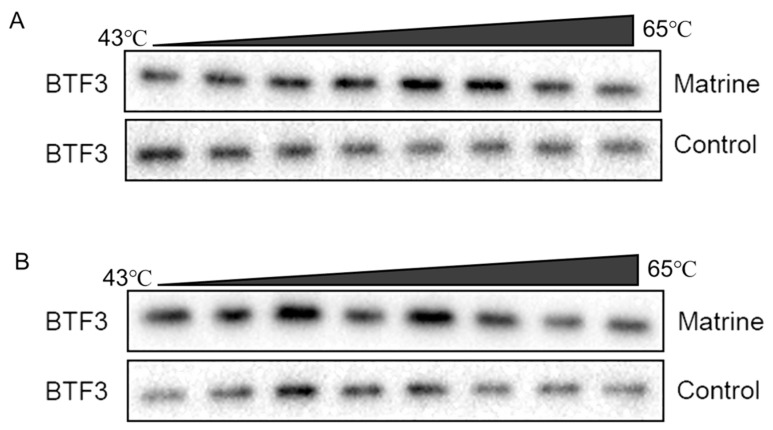
CETSA identifies BTF3 as a target of matrine action. (**A**): CHMm CETSA identifies; (**B**): CHMp CETSA identifies.

The protein becomes more stable and less prone to destruction when the ligand attaches to the target protein precisely. The results showed that the stability of the BTF3 protein of CHMm and CHMp cells gradually decreased with increasing temperatures, and the BTF3 protein of the matrine-treated group was more stable compared to the control group (Figure 9A,B). This indicates that matrine could enhance the thermal stability of BTF3 protein and it was able to bind to the BTF3 protein, indicating that the BTF3 protein has the action of matrine and was the target of matrine.

### 2.10. Effects of Matrine on Btf3 Gene in Canine Mammary Tumor Cells

**Figure 10:**
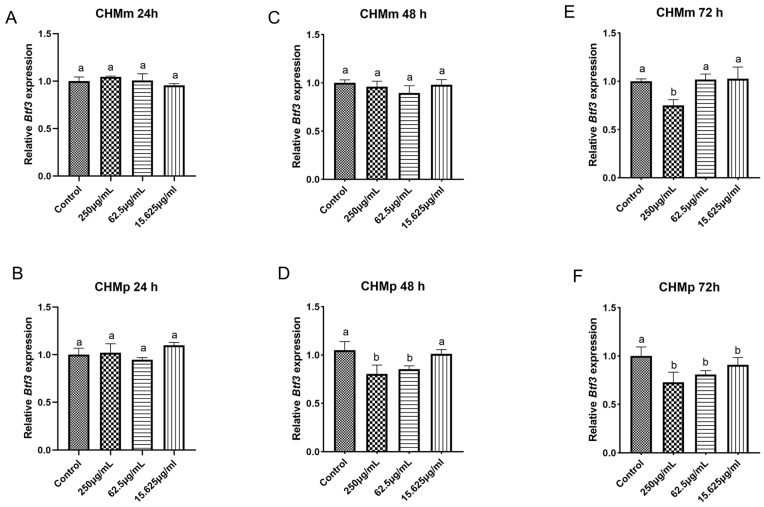
qPCR validation of the effects of matrine on Btf3 mRNA expression in canine mammary tumor cells. (**A**): Matrine action on CHMm cells for 24 h; (**B**): Matrine action on CHMp cells for 24 h; (**C**): matrine action on CHMm cells for 48 h; (**D**): Matrine action on CHMp cells for 48 h; (**E**): Matrine action on CHMm cells for 72 h; (**F**): matrine action on CHMp cells for 72 h. Significance assessed with one-way ANOVA, different letters indicate a significant difference (*p* < 0.05).

The CHMm and CHMp cells were treated with 250 μg/mL, 62.5 μg/mL, and 15.625 μg/mL of matrine, and the results showed that, compared with the control group, there was no significant difference in the expression of *Btf3* gene in CHMm and CHMp at 24 h in different doses of matrine (Figure 10A,B); at 48 h, the expression of *Btf3* gene was significantly reduced in the groups of CHMp 250 μg/mL and 62.5 μg/mL, but there was no significant difference in CHMm (Figure 10C,D); at 72 h, the expression of the *Btf3* gene was dramatically reduced in different groups of CHMp, while the expression of the *Btf3* gene in CHMm was significantly reduced at 250 μg/mL matrine concentration (Figure 10E,F).

### 2.11. Effects of Matrine on BTF3 Protein in Canine Mammary Tumor Cells

**Figure 11:**
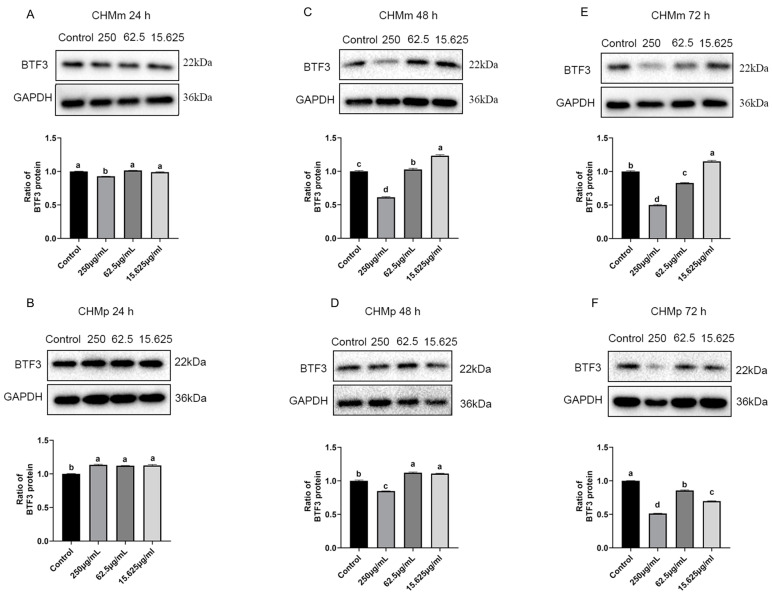
Effects of matrine on BTF3 protein in canine mammary tumor cells. (**A**): Matrine action on CHMm cells for 24 h; (**B**): Matrine action on CHMp cells for 24 h; (**C**): Matrine action on CHMm cells for 48 h; (**D**): Matrine action on CHMp cells for 48 h; (**E**): matrine action on CHMm cells for 72 h; (**F**): Matrine action on CHMp cells for 72 h. Significance assessed with one-way ANOVA, different letters indicate a significant difference (*p* < 0.05).

The CHMm and CHMp cells were treated with 250 μg/mL, 62.5 μg/mL, and 15.625 μg/mL of matrine, and the results showed that compared with the control group, there was no significant difference in the expression of BTF3 protein in CHMp, the expression of BTF3 protein in CHMm was significantly decreased at 250 μg/mL matrine concentration at 24 h (Figure 11A,B); at 48 h the expression of BTF3 protein in CHMm and CHMp was significantly decreased at 250 μg/mL matrine concentration (Figure 11C,D); at 72 h the expression of BTF3 protein in CHMm was significantly decreased at 250 μg/mL and 62.5 μg/mL matrine concentrations(Figure 11E), and the expression of BTF3 protein in CHMp was significantly decreased at 250 μg/mL, 62.5 μg/mL, and 15.625 μg/mL matrine concentrations, respectively (Figure 11F). These results indicated that BTF3 was abundantly expressed in canine mammary tumor cells, and when matrine was applied to the cells, the expression of BTF3 protein was dropped as the action time increased.

## 3. Discussion

Human breast cancer develops spontaneously, thus, there is a need for alternative, yet complementary, models that can better replicate the features of human breast cancer to better understand the molecular and clinical complexities of the disease to aid in the development of new therapeutic strategies [29]. Humans and canines of all ages are at risk of developing mammary tumors in their bodies, and unlike gene-edited animal models, canine tumors originate spontaneously in their natural environment with an intact immune system [30]. Meanwhile, canine mammary tumors are the most prevalent kind of canine tumors, and with more in-depth studies on their molecular features, canine mammary tumors may be utilized as a study model for human breast cancer [31,32].

In this study, we used canine mammary tumor cell lines which were isolated and cultured from the primary lesions and metastatic lesions of 12-year-old female mongrels with mammary tumors. These cell lines efficiently conserved the clinical characteristics of breast tumor primary and metastatic lesions under the same species, and they functioned as a good cellular model for the following search for therapeutic targets.

Matrine, as an active small molecule isolated from legume Sophora flavescens Aiton and other Chinese herbs, has the advantage of a well-defined chemical structure which facilitates the modification and optimization of its structural formula for the research and development of new drugs in the later stage of development [33]. We evaluated the effects of matrine on canine mammary tumor cell lines in vitro. To ensure that normal cells are protected from the toxic effects brought about by the drug, first the drug should be administered to canine mammary epithelial cells for safety screening. The safety concentration experiment was performed on canine primary mammary epithelial cells. Primary mammary epithelial cells were obtained directly from the fresh milk of nursing dogs, and after responding to changes in the in vitro environment, the separated cells restored their proliferation potential and could be used in a drug safety concentration experiment. In canine primary mammary epithelial cells, the maximum non-cytotoxic concentration of matrine and biotin-labeled matrine probe were 250 μg/mL and 500 μg/mL, respectively, concentrations at which both matrine and biotin-labeled matrine probe inhibited the proliferation of CHMm and CHMp. As a result, we have hypothesized that matrine may precisely attach to particular targets of canine mammary tumor cells and elicits anti-cancer effects.

Traditional Chinese medicinal active components form the basis of these effects and are critical to understanding the mechanism of pharmacological action through which binding to specific targets in the body occurs [34,35]. Modifying small molecules into active probes, looking for drug targets and researching drug signaling networks lay the groundwork for improvement and innovation in molecularity targeted therapeutics in traditional Chinese medicine. Cucurbitacin B directly targets IGF2BP1 and covalently binds to cysteine residue 253 of its KH structural domain to promote protein denaturation, according to the development of a molecular probe for its action [36]. To identify the direct target of anti-neuroinflammatory action of wild horse cholestyramine B, a biotin-modified EB molecular probe was constructed and screened using human proteomic microarrays to identify USP7, which is an anti-inflammatory pathway that is activated by EB by inducing a conformational change in the USP7 HUBL region [37]. In this study, we used a biotin-labeled matrine probe and mass spectrometry after co-incubation with canine mammary tumor cell lysate, and GO and KEGG enrichment revealed that the potential target of matrine in canine mammary tumor cells was primarily related to the ribosome pathway, which involves the translation of RNA into protein. Genecards is a comprehensive database that combines the resources of databases such as genomics, transcriptomics, proteomics, and other databases, which can be used to acquire the target of action associated with the condition using disease key words [38]. The Genecards database combined with silver nitrate staining technique was used to screen the target-BTF3, which is a target of matrine that directly interacts with CHMm and CHMp from the potential targets obtained from the ABPP screening.

Based on the principle of increased heat stability of proteins when therapeutic small molecules attach to their direct targets [39], in this study, we have used CETSA and western blot to confirmed the in vitro binding specificity of BTF3 to matrine, and compared changes in thermal stability of target proteins before and after drug action to confirm small molecule binding to proteins. As the temperature increased, the degradation of BTF3 protein decreased in the group with the addition of matrine. Following that, the use of a biotin-labeled matrine probe combined with the streptavidin magnetic bead “target fishing” technique revealed that matrine competitively binds to the BTF3 protein, and molecular docking revealed that matrine can form a hydrogen bond with the THR-89 structural domain of BTF3, which may be the specific binding region of matrine and BTF3, and which can be confirmed in the following stages.

BTF3, which is an essential factor for the initiation of transcription, was isolated from Hela cells and can bind to RNA polymerase II, regulating gene transcription [40]. Evolution is conserved across various kinds of cells and organisms [41]. BTF3 has been reported to be highly linked with many cancers. It has been shown that transfection of BTF3 siRNA into HCT116 cells reduces cell viability, induces apoptosis, blocks the cell cycle, and attenuates tumorigenicity of colorectal cancer cells by decreasing the activities of MAD2L2, MCM3, and PLK147 [42]. Kahweol-treated cells showed a significant decrease in cell viability, an increase in nuclear condensation, and induction of apoptosis through ERK-mediated signaling pathways in a dose- and time-dependent manner, as well as the expression of the BTF3 gene and apoptosis-associated proteins involved in cell cycle regulation [43]. There has been no reported link between matrine and BTF3, so we investigated the changes in BTF3 after matrine action on canine mammary tumor cells from the perspective of genes and proteins. At the gene level, we have discovered that the expression level of the BTF3 gene decreases insignificantly under the action of a short period, and it decreases significantly under the action of a long period. The BTF3 protein began to decrease in a short time under the action of matrine, and it can be hypothesized that the reduction of protein is not due to the reduction in the gene; on the contrary, with the prolongation of the action time of matrine, the protein decreases to a certain extent and will inhibit the expression of the gene, and with the reduction in the expression of the BTF3 protein, the proliferation of the CHMm and CHMp cells is inhibited. These findings laid a foundation for the in vivo experiments afterward, and at the same time, provide the theoretical basis for the targeting of the treatment of matrine in human breast cancer.

## 4. Materials and Methods

### 4.1. Cell Culture

CHMm and CHMp cell lines were gifted by Prof. Yun Liu from Northeast Agricultural University, and both were isolated and cultured from primary and metastatic lesions of 12-year-old female mongrel dogs with mammary tumors. The cells were cultured in DMEM high-glucose medium containing (Thermo Fisher Scientific, Waltham, MA, USA) 10% fetal bovine serum (Cell Max, Beijing, China) and incubated in an incubator 5% CO_2_ at 37 °C. Upon reaching 80% confluency, cells were trypsinized with 0.05% trypsin (Solarbio, Beijing, China) and subcultured.

### 4.2. Maximum Safe Concentration Detection

Our lab has successfully isolated canine primary mammary epithelial cells from canine milk to determine the maximum safe concentration of matrine (National Institutes for Food and Drug Control, Beijing, China) and biotin-labeled matrine probe (The maximum safe concentration for subsequent experiments was based on the concentration that allows over 90% of the cells survive). The matrine and biotin-labeled matrine probe were serially diluted with cell maintenance medium into five gradients, which were 1000 μg/mL, 500 μg/mL, 250 μg/mL, 125 μg/mL, and 62.5 μg/mL, added to a 96-well cell culture plate of canine mammary cells, and a control group (with the addition of cell maintenance medium) was set up and incubated in an incubator (5% CO_2_, 37 °C). Cell lesions were examined on a daily basis. After 72 h, 10 μL of the Cell Counting Kit-8 (Boster, Wuhan, China) solution was added to each well of the plate, carefully so as not to initiate bubbles in the wells, and further incubated for 1 h at 37 °C. A microplate reader was used to determine the OD value at 450 nm. The OD values obtained were used to determine cell survival.

### 4.3. Cell Proliferation Assay

Matrine and biotin-labeled matrine probe were serially diluted two-fold with cell maintenance medium and added to 96-well cell culture plates of CHMm and CHMp cells, and a cell control group (with the addition of cell maintenance medium) was set up and incubated for 24 h, 48 h, and 72 h in an incubator (5% CO_2_, 37 °C). Then, 10 μL of the CCK-8 solution was added to each well of the plate, carefully so as not to initiate bubbles in the wells, and further incubated for 1 h at 37 °C. A microplate reader was used to determine the OD value at 450 nm. The OD values obtained were used to determine the proliferation rate of matrine and biotin-labeled matrine in the CHMm and CHMp cells.

### 4.4. SEM and TEM Analysis

CHMm and CHMp were inoculated in six-well cell culture plates and incubated in an incubator (5% CO_2_ 37 °C) for 36 h with the addition of medium containing varying concentrations of matrine while cell controls were set up. Prefixed with a 3% glutaraldehyde, the cell samples were then postfixed in 1% osmium tetroxide, a portion of cell samples were treated conductively and observed by JSM-IT700HR Scanning Electron Microscope; the remaining cell samples were dehydrated stepwise with acetone, infiltrated in Epon 812 for a while longer, and embedded. The semithin sections were stained with methylene blue and Ultrathin sections were cut with a diamond knife and stained with uranyl acetate and lead citrate. Sections were examined with a JEM-1400-FLASH Transmission Electron Microscope.

### 4.5. Pull-Down

The CHMm and CHMp were inoculated in six-well cell culture plates and incubated in an incubator with 5% CO_2_ at 37 °C. After reaching 90% cell density, the cells were taken out from incubator and washed three times with pre-cooled PBS, centrifuged at 3000 rpm for 5 min each time, and cell lysates (RIPA (Boster, Wuhan, China): protease inhibitor (Boster, Wuhan, China): phosphatase inhibitor (Boster, Wuhan, China) = 100:1:1) were added. The cells were lysated on ice and then centrifuged at 4 °C for 10 min at 12,000 rpm, and the supernatant was the total cellular protein. Following extraction of the total cell protein, the protein concentration was measured by using a BCA kit (Beyotime, Shanghai, China) and corrected to approximately 3 mg/mL. The assay was divided into two groups: the competition group included matrine in the lysate for incubation, after the incubation was completed, the biotin-labeled matrine probe was added, followed by the addition of streptavidin magnetic beads (PuriMag, Xiamen, China); and the probe group added biotin-labeled matrine probe for incubation, followed by the addition of streptavidin magnetic beads. Finally, the magnetic beads were recovered by a magnetic rack and washed with cell lysates and then enzymatically digested with trypsin, and the peptides were desalted after enzymatic digestion, followed by the detection of the protein profiles of the samples using the liquid mass spectrometry (LMS) system composed of an Easy-nLC 1200 ultra-high-performance liquid tandem coupled with a Q Exactive high resolution mass spectrometer (HRMS), and the protein profile of the samples was analyzed by using Proteome Discover 2.5 software (Thermo Scientific, Waltham, MA, USA) for protein characterization of samples from the competition and probe groups of canine mammary tumor cells.

### 4.6. GO and KEGG Analysis

The potential targets of matrine screened by the mass spectrometer in CHMm and CHMp were imported into the DAVID database (https://david.ncifcrf.gov/ (accessed on 1 August 2023)) [44]. GO and KEGG analyses were performed, and the top 10 results were mapped to predict the biological process, cellular component, molecular function, and related pathways of potential targets of matrine action in canine mammary tumor cells.

### 4.7. PPI Network Construction

After accessing the Genecards database (https://www.genecards.org/ (accessed on 1 August 2023)) [45], the keyword “canine mammary tumor” was used to obtain related targets (Table S1) and intersect the potential targets of matrine to obtain the disease targets related to “canine mammary tumors”; accessing the STRING database (https://string-db.org/ (accessed on 1 August 2023)) [46], the targets obtained from screening were imported to obtain the target interactions map, exported as TSV file and imported into cytoscape3.7.2 software (https://cytoscape.org/ (accessed on 1 August 2023)) for visual analytics. The color saturation and size of the circles reflected the degree value of targets, which was used to screen the key targets.

### 4.8. SDS-PAGE Silver Nitrate Staining

After completing the pull-down of CHMm and CHMp cells, the experiment was divided into four groups: protein lysate group, control group with blank streptavidin magnetic beads, biotin-labeled matrine probe, and biotin-labeled matrine probe + matrine group. We added 5×Denaturing Buffer 95 °C denaturation for 10 min to ensure that the proteins were entirely separated from the streptavidin magnetic beads, followed by SDS-PAGE gel (Boster, Wuhan, China) electrophoresis. The gel was then washed, fixed, eluted, sensitized, silver stained, and developed using silver stain sits (Coolaber, Beijing, China) to observe the specific bands. The entire silver staining nitrate process was carried out in a horizontal shaking machine with a rotational speed of 60 rpm, the gel was placed in the termination solution, and photographed after the silver nitrate staining was completed.

### 4.9. Molecular Docking

We accessed the PubChem database (https://pubchem.ncbi.nlm.nih.gov/ (accessed on 1 August 2023)) to download the structural data file (SDF) structural formula of matrine, which was then transformed to Mol2 structural formula using Openbable; we then accessed the RCSB Protein Data Bank (https://www.rcsb.org/ (accessed on 1 August 2023)) to download the PDB structure of BTF3 (Uniprot ID: P20290) [47]. Then, the BTF3 protein and matrine were imported into AutodockToolsl.5.6 [48] for semi-flexible docking, and we selected the conformation with the lowest binding energy after docking was complete and exported, and we used PyMol for visual mapping.

### 4.10. CETSA

CHMm and CHMp were inoculated in six-well cell culture plates, and when the cell density reached 90%, the total cell protein was extracted. The cell lysate was divided into two parts, and matrine was added to one of them and incubated at 4 °C for 4 h. Subsequently, the cell lysate after incubation was divided equally into eight 200 μL tubes and heated sequentially from 43 °C to 65 °C with a gradient of 3 min each time. After the heating, the tubes were incubated for 3 min at room temperature, followed by the addition of 5× loading Buffer to denature the protein at 95 °C for 10 min, and the integrity of the bands was verified by western blot.

### 4.11. qPCR

The CHMm and CHMp were inoculated in six-well cell culture plates and incubated in an incubator (5% CO_2_ 37 °C) for 24 h, 48 h, and 72 h with the addition of medium containing varying concentrations of matrine, and a control group (with the addition of cell maintenance medium) was set up. After the culture was finished, followed by cellular RNA extraction, to thoroughly lysates the cells, RNAiso Plus (Takara, Tokyo, Japan) was applied, followed by chloroform to isolate RNA and isopropanol to precipitate RNA. The RNA was washed with 75% alcohol to remove impurities, transcription kits (Vazyme, Nanjing, China) were applied to remove gDNA and reverse transcribed to obtain cDNA. SYBR Green qPCR Master Mix (Selleck, Houston, TX, USA) fluorescence quantitative reagent and ABI 7500 Real-Time PCR system were used for PCR tests, which included initial denaturation at 95 °C 5 min, denaturation at 95 °C 15 s, annealing and extension at 60 °C 1 min, denaturation, annealing and extension for a total of 40 cycles, and then after 95 °C 15 s, 60 °C 1 min, 95 °C 30 s, and 60 °C 15 s were analyzed for the melting curves, and the GAPDH internal reference gene was used to determine the expression of associated genes. The primer sequences are shown in (Table 2) below.

### 4.12. Western Blot

The CHMm and CHMp were inoculated overnight in six-well cell culture plates and incubated for 24 h, 48 h, and 72 h with the addition of medium containing varying concentrations of matrine, and a control group (with the addition of cell maintenance medium) was set up. After the culture was finished, cells were collected in EP tubes using cell scrapers, and total cellular proteins were extracted using the same method as in 2.5, and protein concentrations were determined using a BCA kit, followed by SDS-PAGE separation of the protein samples, transferring the proteins to poly (vinylidene fluoride) PVDF membranes, closing them with 5% skim milk for 2 h, adding a 1:500 dilution of mouse antibody-BTF3 (Santa Cruz Biotechnology, Santa Cruz, CA, USA) solution for overnight treatment, GAPDH (Proteintech, Wuhan, China) was used as an internal reference, and then adding HRP conjugated affiniPure goat anti-mouse IgG (Boster, Wuhan, China) for 1 h. After darkroom exposure and film scanning, image J 1.42q software was used for analysis and processing.

### 4.13. Data Analysis

All data were expressed as mean ± standard deviation (Mean ± SEM). Data analysis was performed using one-way ANOVA in GraphPad Prism 8 software, different letters indicate a significant difference (*p <* 0.05).

## 5. Conclusions

It was concluded from the present study that the maximum safe concentrations of matrine and biotin-labeled matrine probe in canine mammary epithelial cells were 250 μg/mL and 500 μg/mL. Matrine had a time-dependent proliferation inhibitory effect on CHMm and CHMp cells within a safe concentration range and CETSA findings indicated that matrine could increase the heat stability of BTF3 protein; biotin-labeled matrine probe with CHMm and CHMp cells at safe concentrations had a substantial impact, indicated that BTF3 was abundantly expressed in canine mammary tumor cells. The expression of BTF3 protein was dropped as the action time increased, and the growth rate of canine mammary tumor cells was slowed.

## Figures and Tables

**Table 1:** Core targets of matrine-acting canine mammary tumor cells.

Gene	kDa	Description	Matrine Combined Score	
			CHMm	CHMp
BTF3	22.1	Basic Transcription Factor 3	7.4	7.2
MYH9	226.2	Myosin Heavy Chain 9	6.5	2.8
KPNB1	97.2	Karyopherin Subunit Beta 1	1.7	5.9
EIF3D	65.4	Eukaryotic Translation Initiation Factor 3 Subunit D	2.2	2.1
RBM39	58.8	RNA-binding motif protein 39	2.0	1.7

**Table 2:** Primer sequences.

Gene	Sequence	Product Length
*Btf3*	F: TGCGCTCCAACAAGATGAAAG	105 bp
	R: TCTTCTTTCGGCGAGCAGTT	
*Gapdh*	F: GATCCCGCCAACATCAAATG	166 bp
	R: TCACGCCCATCACAAACATG	

## Data Availability

All data supporting the findings of this study as well as Supplementary Materials are available within the paper and published online.

## Supplementary Materials

The following supporting information can be downloaded at: https://www.mdpi.com/article/10.3390/ijms25010540/s1.

## Abbreviations

SEM	Scanning electron microscopy
TEM	Transmission electron microscopy
ABPP	Activity-based protein profiling
CCK-8	Cell Counting Kit-8
CETSA	Cellular thermal shift assay

## References

[1] Mattiuzzi C., Lippi G. (2019). Current Cancer Epidemiology. J. Epidemiol. Glob. Health.

[2] Giaquinto A.N., Sung H., Miller K.D., Kramer J.L., Newman L.A., Minihan A., Jemal A., Siegel R.L. (2022). Breast Cancer Statistics, 2022. CA A Cancer J. Clin..

[3] Aleskandarany M.A., Vandenberghe M.E., Marchiò C., Ellis I.O., Sapino A., Rakha E.A. (2018). Tumour Heterogeneity of Breast Cancer: From Morphology to Personalised Medicine. Pathobiol. J. Immunopathol. Mol. Cell. Biol..

[4] Vogell A., Evans M.L. (2019). Cancer Screening in Women. Obstet. Gynecol. Clin. N. Am..

[5] Sung H., Ferlay J., Siegel R.L., Laversanne M., Soerjomataram I., Jemal A., Bray F. (2021). Global Cancer Statistics 2020: GLOBOCAN Estimates of Incidence and Mortality Worldwide for 36 Cancers in 185 Countries. CA A Cancer J. Clin..

[6] Mintz R.L., Gao M.A., Lo K., Lao Y.H., Li M., Leong K.W. (2018). CRISPR Technology for Breast Cancer: Diagnostics, Modeling, and Therapy. Adv. Biosyst..

[7] Loibl S., Poortmans P., Morrow M., Denkert C., Curigliano G. (2021). Breast cancer. Lancet.

[8] Swain S.M., Miles D., Kim S.B., Im Y.H., Im S.A., Semiglazov V., Ciruelos E., Schneeweiss A., Loi S., Monturus E. (2020). Pertuzumab, trastuzumab, and docetaxel for HER2-positive metastatic breast cancer (CLEOPATRA): End-of-study results from a double-blind, randomised, placebo-controlled, phase 3 study. Lancet. Oncol..

[9] Long X., Nephew K.P. (2006). Fulvestrant (ICI 182,780)-dependent interacting proteins mediate immobilization and degradation of estrogen receptor-alpha. J. Biol. Chem..

[10] Fidler I.J. (2003). The pathogenesis of cancer metastasis: The ‘seed and soil’ hypothesis revisited. Nat. Rev. Cancer.

[11] Huber K.E., Carey L.A., Wazer D.E. (2009). Breast cancer molecular subtypes in patients with locally advanced disease: Impact on prognosis, patterns of recurrence, and response to therapy. Semin. Radiat. Oncol..

[12] Jin L., Han B., Siegel E., Cui Y., Giuliano A., Cui X. (2018). Breast cancer lung metastasis: Molecular biology and therapeutic implications. Cancer Biol. Ther..

[13] Wu Q., Li J., Zhu S., Wu J., Chen C., Liu Q., Wei W., Zhang Y., Sun S. (2017). Breast cancer subtypes predict the preferential site of distant metastases: A SEER based study. Oncotarget.

[14] Alvarez C.E. (2014). Naturally occurring cancers in dogs: Insights for translational genetics and medicine. ILAR J..

[15] Rowell J.L., McCarthy D.O., Alvarez C.E. (2011). Dog models of naturally occurring cancer. Trends Mol. Med..

[16] Sleeckx N., de Rooster H., Veldhuis Kroeze E.J., Van Ginneken C., Van Brantegem L. (2011). Canine mammary tumours, an overview. Reprod. Domest. Anim. Zuchthyg..

[17] Kim T.M., Yang I.S., Seung B.J., Lee S., Kim D., Ha Y.J., Seo M.K., Kim K.K., Kim H.S., Cheong J.H. (2020). Cross-species oncogenic signatures of breast cancer in canine mammary tumors. Nat. Commun..

[18] Zhang H., Chen L., Sun X., Yang Q., Wan L., Guo C. (2020). Matrine: A Promising Natural Product With Various Pharmacological Activities. Front. Pharmacol..

[19] Sun N., Zhang H., Sun P., Khan A., Guo J., Zheng X., Sun Y., Fan K., Yin W., Li H. (2020). Matrine exhibits antiviral activity in a PRRSV/PCV2 co-infected mouse model. Phytomed. Int. J. Phytother. Phytopharm..

[20] Chu Y., Jing Y., Zhao X., Wang M., Zhang M., Ma R., Ma W., Lv Y., Zhu L. (2021). Modulation of the HMGB1/TLR4/NF-κB signaling pathway in the CNS by matrine in experimental autoimmune encephalomyelitis. J. Neuroimmunol..

[21] Niu H., Zhang Y., Wu B., Zhang Y., Jiang H., He P. (2014). Matrine induces the apoptosis of lung cancer cells through downregulation of inhibitor of apoptosis proteins and the Akt signaling pathway. Oncol. Rep..

[22] Zhou N., Li J., Li T., Chen G., Zhang Z., Si Z. (2017). Matrine-induced apoptosis in Hep3B cells via the inhibition of MDM2. Mol. Med. Rep..

[23] Peng X., Zhou D., Wang X., Hu Z., Yan Y., Huang J. (2016). Matrine Suppresses Proliferation and Invasion of SGC7901 Cells through Inactivation of PI3K/Akt/uPA Pathway. Ann. Clin. Lab. Sci..

[24] Zhou B.G., Wei C.S., Zhang S., Zhang Z., Gao H.M. (2018). Matrine reversed multidrug resistance of breast cancer MCF-7/ADR cells through PI3K/AKT signaling pathway. J. Cell. Biochem..

[25] Ren L., Mo W., Wang L., Wang X. (2020). Matrine suppresses breast cancer metastasis by targeting ITGB1 and inhibiting epithelial-to-mesenchymal transition. Exp. Ther. Med..

[26] Ziegler S., Pries V., Hedberg C., Waldmann H. (2013). Target identification for small bioactive molecules: Finding the needle in the haystack. Angew. Chem. (Int. Ed. Engl.).

[27] Pichler C.M., Krysiak J., Breinbauer R. (2016). Target identification of covalently binding drugs by activity-based protein profiling (ABPP). Bioorganic Med. Chem..

[28] Zhang T., Li J., He Y., Yang F., Hao Y., Jin W., Wu J., Sun Z., Li Y., Chen Y. (2018). A small molecule targeting myoferlin exerts promising anti-tumor effects on breast cancer. Nat. Commun..

[29] Kwon J.Y., Moskwa N., Kang W., Fan T.M., Lee C. (2023). Canine as a Comparative and Translational Model for Human Mammary Tumor. J. Breast Cancer.

[30] Pinho S.S., Carvalho S., Cabral J., Reis C.A., Gärtner F. (2012). Canine tumors: A spontaneous animal model of human carcinogenesis. Transl. Res. J. Lab. Clin. Med..

[31] Khanna C., Lindblad-Toh K., Vail D., London C., Bergman P., Barber L., Breen M., Kitchell B., McNeil E., Modiano J.F. (2006). The dog as a cancer model. Nat. Biotechnol..

[32] Nguyen F., Peña L., Ibisch C., Loussouarn D., Gama A., Rieder N., Belousov A., Campone M., Abadie J. (2018). Canine invasive mammary carcinomas as models of human breast cancer. Part 1: Natural history and prognostic factors. Breast Cancer Res. Treat..

[33] Sun X.Y., Jia L.Y., Rong Z., Zhou X., Cao L.Q., Li A.H., Guo M., Jin J., Wang Y.D., Huang L. (2022). Research Advances on Matrine. Front. Chem..

[34] Huo X., Gu Y., Zhang Y. (2022). The discovery of multi-target compounds with anti-inflammation activity from traditional Chinese medicine by TCM-target effects relationship spectrum. J. Ethnopharmacol..

[35] Wang Y., Hu B., Feng S., Wang J., Zhang F. (2020). Target recognition and network pharmacology for revealing anti-diabetes mechanisms of natural product. J. Comput. Sci..

[36] Liu Y., Guo Q., Yang H., Zhang X.W., Feng N., Wang J.K., Liu T.T., Zeng K.W., Tu P.F. (2022). Allosteric Regulation of IGF2BP1 as a Novel Strategy for the Activation of Tumor Immune Microenvironment. ACS Cent. Sci..

[37] Zhang X.W., Feng N., Liu Y.C., Guo Q., Wang J.K., Bai Y.Z., Ye X.M., Yang Z., Yang H., Liu Y. (2022). Neuroinflammation inhibition by small-molecule targeting USP7 noncatalytic domain for neurodegenerative disease therapy. Sci. Adv..

[38] Harel A., Inger A., Stelzer G., Strichman-Almashanu L., Dalah I., Safran M., Lancet D. (2009). GIFtS: Annotation landscape analysis with GeneCards. BMC Bioinform..

[39] Li Z., Zhang Y., Chen L., Li H. (2018). The dietary compound luteolin inhibits pancreatic cancer growth by targeting BCL-2. Food Funct..

[40] Zheng X.M., Black D., Chambon P., Egly J.M. (1990). Sequencing and expression of complementary DNA for the general transcription factor BTF3. Nature.

[41] Zhang D.Z., Chen B.H., Zhang L.F., Cheng M.K., Fang X.J., Wu X.J. (2017). Basic Transcription Factor 3 Is Required for Proliferation and Epithelial-Mesenchymal Transition via Regulation of FOXM1 and JAK2/STAT3 Signaling in Gastric Cancer. Oncol. Res..

[42] Liu Q., Wu J., Lu T., Fang Z., Huang Z., Lu S., Dai C., Li M. (2019). Positive expression of basic transcription factor 3 predicts poor survival of colorectal cancer patients: Possible mechanisms involved. Cell Death Dis..

[43] Jeon Y.J., Bang W., Cho J.H., Lee R.H., Kim S.H., Kim M.S., Park S.M., Shin J.C., Chung H.J., Oh K.B. (2016). Kahweol induces apoptosis by suppressing BTF3 expression through the ERK signaling pathway in non-small cell lung cancer cells. Int. J. Oncol..

[44] Sherman B.T., Hao M., Qiu J., Jiao X., Baseler M.W., Lane H.C., Imamichi T., Chang W. (2022). DAVID: A web server for functional enrichment analysis and functional annotation of gene lists (2021 update). Nucleic Acids Res..

[45] Stelzer G., Rosen N., Plaschkes I., Zimmerman S., Twik M., Fishilevich S., Stein T.I., Nudel R., Lieder I., Mazor Y. (2016). The GeneCards Suite: From Gene Data Mining to Disease Genome Sequence Analyses. Curr. Protoc. Bioinform..

[46] Szklarczyk D., Kirsch R., Koutrouli M., Nastou K., Mehryary F., Hachilif R., Gable A.L., Fang T., Doncheva N.T., Pyysalo S. (2023). The STRING database in 2023: Protein-protein association networks and functional enrichment analyses for any sequenced genome of interest. Nucleic Acids Res..

[47] Wang L., Zhang W., Wang L., Zhang X.C., Li X., Rao Z. (2010). Crystal structures of NAC domains of human nascent polypeptide-associated complex (NAC) and its αNAC subunit. Protein Cell.

[48] Forli S., Huey R., Pique M.E., Sanner M.F., Goodsell D.S., Olson A.J. (2016). Computational protein-ligand docking and virtual drug screening with the AutoDock suite. Nat. Protoc..

