# Supplementary material for: Matrine Targets BTF3 to Inhibit the Growth of Canine Mammary Tumor Cells
Source: Int J Mol Sci. 2023 Dec 30;25(1):540. doi: 10.3390/ijms25010540 (PMC10779273; doi:10.3390/ijms25010540)
Supplement: Supplementary file 1 [file ijms-25-00540-s001.zip › Supplementary File S1.pdf]

Table. S1 The target of canine mammary tumor predicted by Genecards

Targets
TP53, APC, PTEN, KIT, BRCA2, PDGFRA, WT1, BRCA1, SMARCA4, TNF, PIK3CA, ERBB2, CDH1, CDKN2A, TP63, KRAS, CTNNB1, H19, EGFR, AKT1, ESR1, BRAF, SMARCB1, MDM2, TBX3, HRAS, CCND1, VHL, DICER1, MEG3, MIR21, MYC, STK11, MEN1, RB1, RET, NF1, VEGFA, CDKN1B, IL6, AR, MTOR, TNFRSF1A, GAS5, CASP8, IDH1, MALAT1, MIR34A, CDC73, FGFR2, NF2, STAT3, CDKN1A, MIR125A, FGFR1, BCL2, XIST, PGR, VIM, MIR145, TNFSF10, TERT, MIR373, IFNG, MUC1, MET, HIF1A, RHOA, CD274, LOC126862571, CCAT1, TNFSF11, MIR200C, KRT19, IL2, SDHA, BAX, TSC2, MIR17, JUN, CDK4, TGFB1, MIR146B, FAS, PTCH1, TP53BP1, MAPK1, MKI67, FASLG, MIR155, IL1B, EGF, NRAS, CXCL8, MMP9, LINC-ROR, MIR200B, CDK2, MIR146A, ITGB1, PTGS2, LATS1, MIR126, MIR203A, PRL, MIR200A, SYP, SRC, KRT7, MIR30A, PTPN11, IL10, MIR29C, CASP3, RAD51, MIR27A, PAX6, MIRLET7C, ENO2, CHGA, KITLG, CD44, FGF3, MIR18A, CERNA3, MMP2, NFKB1, IGF1, ERBB4, SHC1, SST, MIR199B, MIR7-3HG, MIR133B, KDR, LINC01672, FGF2, MIR98, NOTCH1, PIK3R1, IGF1R, DES, MIR320A, PPP2R1A, DNMT1, AXIN2, SCARNA5, MGMT, HULC, RAF1, TGFA, ABCB1, AURKA, PRKAR1A, MIR34C, IDH2, MIR214, PDGFB, MIR451A, MT-CYB, LATS2, SF3B1, HNF1A, MED12, MAP2K1, NR3C1, MIR181A1, ROCK1, PTHLH, PCNA, JAK2, IL4, BIRC5, HGF, MIR181A2, PARP1, IRF1, HMGA2, MIR122, PIK3CG, CAV1, YAP1, CCL2, TNFRSF11B, TMX2-CTNND1, INS, MIR328, BCAR4, ACTC1, CXCR4, FHIT, ICAM1, S100B, MMP1, CD34, MIR101-1, BMPR1A, TNFAIP3, BCL2L1, STAT1, SERPINB5, CYP19A1, MIR206, LINC02605, MAPK8, AFP, BDNF, NOS2, MMP14, SOX9, XPO1, PCAT1, MIR29B2, CD40, MIR181D, ABCC1, TIMP1, PLAU, AREG, CSF1, HSP90AA1, PPARG, MIR494, GFAP, CALCA, MIR29A, RECK, CDK6, CTLA4, SETD2, CXCL12, LINC00511, SPP1, BMP6, IL1A, KRT5, PTK2, MAPK14, SMAD5-AS1, TOP2A, SUFU, COL1A1, CRP, CKS1B, FADD, LEP, KRT18, TRAF6, CSF2, SP1, EDN1, KIF1B, TERC, YY1, CREB1, CASP9, SERPINA3, CDK1, RAC1, TLR4, GLI1, TWIST1, FN1, MAPK3, BDNF-AS, BMP4, TRAF2, ARID1A, BSG, ALB, SLC2A1, MIR93, CALB2, POU5F1, SHH, ABL1, KRT8, CHUK, MIR23A, HDAC1, CDH2, AXIN1, MMP3, FLT1, CD40LG, WNT5A, NGFR, SERPINE1, MIR424, RARA, FOS, CTTN, GSTP1, PLAUR, PDPN, THBS1, HERC2, PRKCD, ZEB1, XIAP, ABCG2, IFI27, CA9, BMP2, NCAM1, MIR136, SMARCE1, KRT14, FLT4, KLK3, IKBKG, PDCD1, GJA1, MAX, MME, CSF3, NPM1, S100A1, IFNA1, MIR193A, GH1, GJB2, TFF1, AIP, CTNNA1, RUNX2, MTA1, GAST, ZEB2, TP53BP2, EGR1, HNF1A-AS1, SOD2-OT1, CASC8, VDR, EZR, ADIPOQ, HMGB1, TLR2, NES, MITF,

SOX2, CTSB, GRP, HPSE, EPHA2, CDKN3, ETV1, LEF1, CYCS, IL1RN, H2AC18, MMP7, SNAI2, CTSD, CCL5, KRT17, CD36, B2M, PRKCA, AXL, TIMP2, CDH3, MIR215, ENO1, GSK3B, IL2RA, DDR1, BMI1, MIR24-1, ANXA5, DHFR, LGALS1, MIR486-1, MMP13, SOD1, MLANA, MIR30B, LGALS3, HNF1B, SPARC, ANO1, IL18, POLD1, ENSG00000277577, TNFRSF17, INHA, FLT3, TRAF3, CASC15, VIP, EDA, TFRC, CADM1, PIP, PECAM1, PCAT2, KLF4, NRP1, MIR375, GRB2, GLI3, POMC, HSPA4, FGF7, CYP1A1, MIR26B, PLK1, IRS1, ENG, HSP90B1, DSCAM-AS1, IL3, SOS1, HLA-DRB1, WNT1, GAPDH, TOP1, CCND2, MST1R, FOXP3, RMRP, HSPA5, CCND3, HSPB1, SNAI1, FGF1, WNT10A, FGF8, CCN2, BIRC3, NTRK2, MIR302D, TKT, ITGA6, MYOG, CD68, CASR, JUP, TPM3, TEK, BCL2L11, ANXA2, SNORD15A, IGF2R, YBX1, NGF, MAPK10, DLC1, SYK, ANGPT2, WNT10B, GHRL, INSR, LINC00673, TYR, MIR493HG, STAT5B, PRKACA, F3, CDC42, TGFB2, ST14, ELAVL1, ETS1, CTNND1, RNY3, TFAP2A, HMOX1, RANBP2, KDM1A, ENSG00000276919, OSM, TFE3, NR1H2, CASC19, NCOA3, CD80, BCOR, IL6ST, CSN2, PGR-AS1, CEACAM1, KMT2D, CASP10, CD4, CEACAM3, MIR130A, HACE1, MYB, ITGAV, CTSK, CAT, TCF3, SMARCA5, TET2, FGF10, EPO, FOLH1, ACTB, MIR24-2, DNMT3B, SQSTM1, TG, TJP1, SH3PXD2A, LMNA, MPO, CLU, EDAR, PRKDC, PAX3, MAP2K2, TNFAIP6, PAK1, MIR27B, HTRA1, LOX, ILK, IFNB1, TRC-GCA24-1, MMP11, ITGB3, PROM1, CXCL10, PTGS1, POSTN, RXRA, LRP6, MAPT, EPB41L3, DKK1, GATA4, PPARA, KNG1, CSNK2A1, CASP7, CYP3A4, MIF, NOS3, ITGA5, BIRC2, MIR381, HDAC2, GNAI2, KCNQ1, ANXA1, NPY, MIR29B1, LIF, FANCI, HSPA1A, IL15, NTS, DDIT3, UBE3A, ALOX5, SDC1, TUBB3, TF, HMGA1, PTPN1, TGIF1, MIR197, HLA-A, TNFAIP8, LDHA, HBEGF, HSPA8, BGLAP, CALD1, MYLK, PIK3R2, ABCC2, JAK1, NDRG1, MIR132, CASP1, TGFB3, DLEU2, PTK2B, ID1, PAX2, MROCKI, FOXC1, CASC9, LALBA, CLDN4, MYOD1, LGALS3BP, GDNF, MIR542, GDF15, STC1, S100A9, IL7, IL13, PTH, RPS27A, GNRH1, MSN, ATF1, TRAF4, ANPEP, VCP, CGB5, PLG, ATP4A, LCN2, ACP5, PTN, GPER1, PIK3CB, IL11, APOE, VTN, ARID2, COMT, ERCC6, MIR100HG, FGF4, TSHR, CCN6, TBX2, GNAQ, PRKD1, MIR30D, TXN, F2RL1, UCHL1, CCK, MIR485, EPOR, TGM2, S100A6, SMARCC2, MYH9, RARS1, SPHK1, SGK1, RBL2, EMSLR, CLTC, BRD4, SNORD44, CCKBR, GAL, IL6R, TLR9, APAF1, GHR, CFTR, AURKB, LTF, OXT, CASP2, GNA11, NANOG, TRA-TGC7-1, TRA-TGC5-1, HSPG2, EDNRA, MIR425, PLAT, CHKA, CD47, LYVE1, RACK1, RBBP4, ALPP, SNORD118, AGER, ELANE, PLCG1, ITGA3, AGTR1, BCAR1, IRF6, CLDN1, BIN1, EPHA3, RBM5, SMARCC1, SCRIB, BAD, EBAG9, BMP7, CSNK1A1, CXCL9, MCM2, CLDN7, BCL6, NUMA1, ACTN4, LIG4, ALCAM, CR2, MDH2, NAMPT, ARAF, RELB, RPS6, HSPD1, NOD2, NCL, REN, MCAM, MSX1, LHCGR, PWAR1, APP, MYH11, MAP3K7, BTRC, ACP3, FLNA, XBP1, NR3C2, KLK10, MB, PTH1R, S100A8, DPP4, SERPINB3,

CGA, TK1, DLG1, SLC25A11, OXTR, FAP, FGF9, RBL1, SLC5A5, CRH, CXADR, CALR, ZEB2-AS1, NTN1, IL4R, MIR302A, WNT3A, ADM, UBE2I, CCR5, CD14, S100A7, CDC25B, ADAR, UBC, MAP3K5, NT5E, USP7, TH, MAP2K4, PRKCZ, STARD13, HSP90AB1, BCR, DKC1, FURIN, EDA2R, VPS35, PLA2G2A, CCL3, HDAC6, LIFR, ITGA2, HAVCR2, GSN, GCGR, CD86, TRE-TTC3-1, PAX9, THBD, ADRB2, PAX5, RHOB, ALOX12, CYP2D6, CSNK2A2, TRIM25, SLC9A1, IGF2BP1, EREG, ELOB, HP, EDN3, LOC110806263, IL24, ITGAL, MAP2K3, SHBG, WNT3, DSP, NUMB, PMEL, SFTA3, GRPR, PTGER4, KRT1, THY1, ARHGEF2, HLA-C, ACE, ENAH, EDNRB, ACACA, VTRNA1-1, IL33, CTBP1, PRC1, PTPN12, SPECC1L, ATP7B, CXCR2, POLR2A, CD9, HUWE1, ELP1, NKX2-5, TBX19, RHOD, F2, TCF7, GCG, SCT, GPX1, ELK1, XRCC4, HIPK2, XDH, IKBKE, YWHAE, PTX3, TUBA1B, EGLN1, MIR605, TOPBP1, TBX5, HSPB2, HAS2, SEPTIN9, ADCY10, LRP1, PTPA, AGR2, SFRP2, IL1RAPL2, TFF3, PRKCE, GSTT1, E2F3, CD163, ANK3, INSL3, MSX2, GLUL, PKD1, OPTN, ITGA4, SERPINA1, DKK3, SAMHD1, G6PC1, APOD, RYR1, DCN, MIR584, COL7A1, LINC01554, PFN1, ACP1, ITGB2, WWC1, VWF, BCORL1, PSEN2, SUMO1, KMT2A, HLA-DQB1, SRGAP1, VIPR1, TRPV4, SELL, GUSB, APOA1, FXYD3, CSN1S1, TRP-AGG2-1, ITGAM, FEN1, MIR551A, RPS19, CYP1A2, AZGP1, DMD, TFEB, ZAP70, MIR340, TRP-AGG2-5, PARD3, ARNT, MBP, ZNF350, ARHGAP35, DSPP, TRP-AGG2-6, TRP-AGG2-2, TRP-AGG2-4, TRP-AGG2-7, SLC2A3, TANK, PODXL, RABL3, SLC16A1, NECTIN4, NHERF1, PSIP1, SLC6A2, CTSL, VCAN, CA12, CDK7, LPO, MST1, HPRT1, G6PD, CTNND2, TRP-AGG2-3, TRP-AGG2-8, COL1A2, TIAM1, WFDC2, VCL, NEDD4, PTOV1, CD79A, SLC2A4, IL5, ROCK2, MEG8, LUC7L2, SP3, CDH11, PITX2, TRB, GPT, PLA2G4A, REL, SLPI, RAB4B-EGLN2, PWAR4, SLC29A1, EPHX1, CCNH, DDX3X, CD63, POLR1C, COL2A1, RNU6-1, JUNB, IL2RG, WNT4, AGO2, IBSP, PA2G4, PEBP1, FUBP1, UBE2D1, MSI1, MIR590, SAA1, SMAD1, RNU6-7, RNU6-8, TES, LRP5, EFNB2, NKTR, TMPRSS2, NET1, LPL, TAB1, AMHR2, DRD2, HCCAT5, MCM7, GBA1, ROBO1, DNAH8, CCL11, SULT1A1, HNRNPK, TLR3, FZD6, COL4A3, MIR379, HPGD, MMP12, SPTBN1, PDX1, CD46, PRKCB, SMURF2, SRSF2, COL4A1, PCSK2, GAB2, RNU6-1-001, RPS6KA1, MMP8, PINX1, SATB2, RNU6-2, CSNK1D, PSCA, IL12A, RNU6-1-003, RNU6-1-004, HNRNPA1, IQGAP1, CAV2, MPL, GLO1, TLR7, RNU6-9, IAPP, TFPI, SRD5A1, SOAT1, MAP2, PPP2CA, CASP6, CCL26, YES1, RDX, RRAS, PBX1, CCNB3, PGRMC1, GPSM2, NUDT6, AQP1, RNU6-1-002, CD2AP, ADA, THPO, EPHA1, OTUB1, ATP2B2, PPY, FYN, TPO, MDK, ATP2A2, CA2, CLDN6, ILF3, BUB3, COLCA1, LLGL1, CCL20, MAML3, MAGED1, PKHD1, TJP2, DDX59, TRA, TBK1, RHO, PSEN1, PHF1, FLT3LG, KPNA2, SLC5A8, IGHE, TBX1, MIR20B, RBBP7, BAG6, COL17A1, PIGR, PSAP, DHX9, PI4KA, COL4A6, PRKAA1, LDLR, NPPB, OPRM1, ABCB4, AKAP13, POU1F1, CXCR1, RBP1, INTS7,

PCSK1, H4C16, TTN, MIR33B, YWHAZ, SAFB2, DUSP19, GGT1, CCR1, KCNMA1, DGCR8, TUBA1A, PSMA1, RIGI, PTGER2, PGK1, AICDA, MIR218-2, TPBG, KRT16, NME2, KISS1R, COX5A, PROX1, PAK4, TFAP2B, P2RY1, IRF4, SYNPO, HOXB7, GALC, TBX18, MAPKAPK2, NPPA, MIR218-1, MT-CO1, COL4A5, LAMA3, PVR, TAGLN, HYAL2, NOS1, NTSR1, UGT2B15, MMP17, SPTLC1, ATP1A1, CRKL, MT-TP, MADCAM1, APOB, PRKCI, IL23A, DNAJB1, GNB3, ATP12A, TCOF1, TFF2, DSG3, CCR4, ABCC11, RXRB, TRPM8, AHNAK, FPGS, LBP, ACTL6A, ZYX, TBX20, CCL4, NOD1, POU2F1, DRD4, F5, GAB1, DSG1, LRP2, ELN, LNPEP, ARHGEF5, CYP2B6, HNRNPM, DCAF1, HNRNPC, SELP, FCGR1A, MGA, TRPV1, ABCC3, COL5A1, RALBP1, ECE1, CCNT1, IL18R1, ST13, RBM19, SLC17A5, POLR2L, COMMD1, SKP1, SLAMF1, RPS29, SLC3A2, HOXA9, NPHS1, ITCH, HPR, PPARD, RLIM, MIR1246, EEF1G, ACTN1, MAZ, CP, NECTIN1, PDIA3, BCHE, YBX3, UBE2D3, LSP1, ABCB11, PARK7, SMC1A, AQP4, MBL2, PMP22, TPX2, FBN1, HPN, CCL15-CCL14, PRDX2, KCNB2, EGLN2, PPIG, CREB3L1, IMPDH2, EDARADD, DBH, CSTA, EPB41, ATP7A, KCTD12, CFL1, CDH17, FKBP4, CASP4, CYP2C9, IVL, AKR1B1, MCM3, DHCR7, CALCR, PKN1, ACHE, COL11A1, AMOT, CDK9, RUVBL1, PRPF8, ZC3H7B, PTGER3, PIK3C2A, MIR133A1, SRY, CACNA1G-AS1, DLX3, MRC2, CD1A, DDB1, KRT4, DEK, RPLP0, CD69, CLDN3, CCL17, TNS4, LRPAP1, PUF60, SRF, PTP4A3, AMOTL1, PSMB5, PRNP, EGLN3, RNF2, MGAT5, CENPF, HSD3B2, THORLNC, SNORD24, AKAP1, TAC1, CLDN2, SLC22A2, PLCB3, ECT2, AGK, LGALS7, YWHAQ, FAT3, CRK, RAN, ST6GAL1, TRPC1, IL9, UBR5, KRIT1, HNRNPU, ITPR3, ALDOA, DYRK1B, GHSR, TBP, DNM2, CCL28, DAG1, TIMP4, VDAC1, MECP2, TPH1, F9, NMBR, LINC00342, IL10RA, FLNB, VAV1, SLC7A11, AMBP, BLZF1, CAMP, PTAFR, ZFPM2, HNRNPL, MANF, KCNJ5, ACLY, GANAB, KCNJ11, CASP14, SMURF1, TRIP12, CAPN1, ABCC4, RCN1, HSD11B1, PKD2, HYOU1, KCNH2, PPP1R9B, COL6A1, TLR5, ILF2, IRF3, SFTPC, CARD16, SF1, TUBG1, ANAPC1, PRKG1, VANGL1, PLEKHA8, SLMAP, CKB, INPPL1, SLC26A4, MAVS, WWP2, UBE2D2, WDR11, CCR3, FAIM2, PABPC1, DDX17, EIF6, RPLP1, KIF16B, AHCY, RNY5, NEU1, NEDD4L, DCT, HNRNPD, ACTA1, AVPR2, ANKRD11, CA6, ALOX15, PDE4A, C1orf35, GJB6, VIRMA, AMPH, ISG15, ROCR, CIRBP, FAS-AS1, UPF1, RBM6, SLC22A5, CCL21, MX1, MIR101-2, AMELX, GFI1, LIMD1, LAMP2, INHBA-AS1, KLHL20, RCVRN, WNT7A, SDCBP, ARHGAP5, LBR, CST3, KLK2, RBM15, TNKS2, ERN1, RRP1B, IRF7, PNN, HSD17B4, KPNB1, ANKRD17, MXD1, MAOA, CSE1L, CHD7, CCL27, P4HB, KIF5B, BAG3, CTNNBIP1, BHLHE40, ADARB1, FSHB, MCF2, CASK, LONP1, TPR, RRBP1, PROP1, HRH2, MC4R, NEK1, CCDC6, LAMA5, PYY, IVNS1ABP, PSMC6, MIR486-2, CAMK2D, SPAG9, SOX9-AS1, SNRNP70, KIAA1671, LBX1, TRPC6, TAP2, STAT2, SLC28A1, SLIT3, RAB25, PLEC, ATP2B4, PRDX5, LIN7C, AVP, MPDZ, ENSG00000288605, MEIS1,

MYH10, CES1, ABCC5, HHIP, CHSY1, TARDBP, RAC3, RBBP5, RNF216, TRAF3IP2, SMC3, TRIM21, CNP, SLC1A2, MAMLD1, ATP1B1, ERBIN, TNNT2, CCL19, RBMX, QKI, HTR2A, CAMK2G, NEDD9, MAPRE2, FCGRT, PHEX, VAV3, ERVW-1, KCNA5, DPEP1, ITGB6, AMOTL2, HTRA2, LDHB, TRRAP, ARHGAP1, AQP5, ADRB3, ARRB2, DERL1, SLC31A1, RBM39, H3C14, IL13RA1, HNRNPUL1, DNASE1, TYK2, NOP16, MAP3K6, P2RY2, NUP98, SFTPB, S1PR1, MARCKS, UBA1, FCER2, MRPL28, FABP12, KDM5A, SLC22A1, UBE2L3, PTMA, TRIP6, PPP2R5C, TBX22, MIR744, HGFAC, ECRG4, HCRT, H3C12, SLIT1, LOC654780, PDCD10, MAP2K7, RAB1A, TBX4, UBA52, LOC102723517, WDR6, ASPH, TRIM32, MARK2, RNU4ATAC, PRKACB, SLC12A2, SOD3, OCLN, P2RX7, EIF4A3, CACNA1C, CEP85L, CRYAA, PDZK1IP1, KDM2B, CNR1, PIK3C3, MCM5, MAP3K20, WNT6, C5AR1, TREX1, GLUD1, PAF1, DCX, LGMN, ARHGEF17, KRT9, LOC109286556, PORCN, ST20, SRGAP3, PKP2, APOH, GCLC, DOCK8, CBLL1, CPSF6, PPP1R13L, SPECC1L-ADORA2A, CDC37, BAG4, CYBB, CPQ, STX1A, ARHGEF4, FCGR3B, NACA, SERPINH1, ERAS, TAGLN2, RBCK1, SLC16A4, FTH1, DNM3, TRIM27, RPS5, FKBP1A, MIR105-1, SMTN, CANX, TBX15, NMB, RLN1, PRRC2A, CYBA, PAK2, MLN, DDX39A, LRAT, GIPC1, PPP6C, COMP, STXBP2, ZFP36L1, FOSB, PPP1CA, DBN1, MCM6, RACGAP1, SPINK5, PROS1, HAS2-AS1, NUP93, COL3A1, HEXIM1, SPTAN1, IL22, PLIN1, EIF3D, CSN3, MMP20, GYPA, FOXQ1, TFAM, GCC2, CEP350, PPA1, PDIA6, SNORD43, NISCH, FUCA1, DCTN1, ACE2, FHL1, NMI, FXYD5, EPIC1, CYFIP1, LSM2, SCN5A, RTRAF, TPI1, TBX6, SLC15A1, EPHX2, CLDN8, PADI2, TLN1, LACTB, APPL1, CD1D, SLC1A3, XRN1, NEMF, DLG4, CAV3, BLVRB, BDKRB2, KLK5, ADAMTS13, OFD1, HNRNPR, ITGAX, NALT1, ANP32A, COMMD9, TLR1, STRA6, CD22, CUBN, VAV2, TPP2, ANK2, ASAP1, HTR3A, CHRM3, PWRN1, CTBP2, TCTN3, PDS5A, SCNN1B, CHRNA3, U2AF2, RAB5A, GNA13, HP1BP3, AKAP8, FBXW11, MYOZ2, CCDC68, PABPC4, TSPEAR, GJA5, TAT, CAND1, AIF1, LINC01587, TCERG1, CUL7, RPN2, UBAP2L, COL4A4, TNKS, LGALS4, SPATA13, HNRNPF, GART, PCGF1, SRSF5, PKP4, HAS1, FLNC, MAP4K4, INA, TCHH, FMR1, AIRE, ATP5F1A, EEF1B2, MT3, GCN1, SERPINC1, CSTB, WWP1, IFNGR1, DDX50, DST, ADORA3, EPS15, CSNK1E, ARHGEF7, BMP1, MMP16, SFTPD, PCK2, PWAR6, DLG3, F8, CACYBP, SEC16A, SERPINA5, CCDC47, CCKAR, RAB11A, HAX1, STRN, IRF5, CNTF, NEFL, PPP1R12A, SEC13, NUP35, AGL, TRD-GTC9-1, SSB, PRPF6, PCNT, SRRT, LAPTM4B, MIB1, ADORA2A, THRAP3, PRKAR2A, PKN3, RHBDD1, PICALM, TASOR, HBB, MAGED2, ATP1B2, MAL2, VASP, SLC1A5, MAGI1, CBS, NMU, CKAP4, IFNAR1, ARHGEF15, GNAI1, SH3KBP1, DYNLL1, PALS1, TICAM1, ASRGL1, DUT, TLR6, DHX37, HMOX2, HADHB, PDCD6, PSME3, EEF1E1, BST2, DNAJB11, WDPCP, RRAGC, PRSS2, IFT88, ATP2B1, RAB10, CBX3, DAPK3, CLEC4A, F7, FLOT1, MYO6,

PRDX3, GPHN, PPP1CC, UGT1A, SNAP25, UBR4, G3BP2, GNAI3, PSMA6, STX3, CAPN2, ANXA6, MAP2K6, MMP25, S100A10, DNAJC9, SUPT5H, DDX21, KIF4A, CDC23, HIF1AN, KTN1, MRPS7, PSMA5, HNRNPAB, GIT1, PRDX4, GUK1, USP39, PALM2AKAP2, SLC5A1, NIPSNAP1, CAVIN1, PPL, ABCC6, SNORA66, RPN1, CPSF1, HCRTR2, PKP3, LCAT, NOP56, C2CD3, SCNN1G, MCTS1, SLCO1C1, CYTH2, LRRC59, ITPR1, BCAP31, ADRB1, SLC8A1, IQCB1, NOL7, PLOD1, TMPRSS11A, RAB7A, STX2, RGN, DLAT, SLC6A6, NASP, COPA, SH3GL1, DLX2, WASL, CADM4, ACAN, PRKCG, API5, PROC, NOP58, KCNN4, CIAO1, SOSTDC1, PPIB, AQP7, HNRNPH3, DIRAS1, ZRANB1, FARSA, CANT1, DCAF7, BTF3, GFER, PLCB1, VIPR2, MYO5B, ANKRD1, RANGAP1, AMBN, PAPPA-AS1, KCNJ10, F11R, MAT2A, KHDRBS3, TBXA2R, CPM, VARS1, BCAM, ATP6V1B2, CYP2J2, TRIM31, PSMC5, LYZ, RPE65, PLOD2, RAB11FIP4, SIGLEC7, GAA, SHCBP1, NUP88, CLDN5, SUB1, TPP1, VAC14, SGPL1, RBMS3, GRHL3, TGM1, CD209, LNX1, MCPH1, SOS2, IPO5, CCAR1, AQP3, CTSG, LAMTOR5, CES2, PARVA, MRAP2, EIF3G, LSM14A, DSC3, AQP2, TNNI3, SRP72, GLB1, SYMPK, ST7-OT3, VAMP7, CLDN10, ARID3B, SFTPA1, GSPT1, RAPH1, SEC11A, FAM3C, PDE5A, MLLT3, PRPF3, IDUA, ITIH4, SNX6, UTRN, AKAP8L, SF3A1, HSD17B10, OTC, RPL10L, ZNF326, GKN1, TCN1, RALB, LINC01191, RANBP1, STIP1, IL19, FCER1A, ADD1, PYCR1, AP1B1, CNTFR, MIR299, SCNN1A, NUAK2, DHX30, LYAR, MPP2, DLG2, CPLANE1, PSMA7, RBFOX2, RNF128, SRGN, PI4KB, ASH2L, LMNB2, RBP4, LARP1, CEP170, ARPC1B, KIF3A, MR1, PARD6A, RYR2, RNF217-AS1, CS, PSMD12, COPB1, NBPF1, RANBP9, GBF1, GIGYF2, S1PR2, SEPTIN2, TNKS1BP1, NBPF12, ARF1, MIR758, EEA1, SIL1, SLCO1B3, ADH5, USP34, SSR2, BEST1, PABPN1, WDR35, EVPL, CSDE1, NEURL4, SMOC2, ZNF398, RRAGB, KIF7, AOC3, RPL22L1, ANXA11, RAB14, LRPPRC, SRP14, ZC3HAV1, ARHGAP29, POLR2B, ARF4, KCNJ3, JPT2, MIR208B, SNRPA, PDE3A, SCTR, CAPG, RAE1, LUM, HAVCR1, TMEM33, STAU1, NUP62, TRIP10, P2RX5-TAX1BP3, OCRL, SRP68, M6PR, SLC39A10, OPRK1, ESD, LLGL2, ALOX5AP, TRG, CLCN7, NCK1, EPPK1, CCDC88A, MAOB, THOC1, SRRM1, PDP1, ATXN2L, PAICS, SCLT1, SPSB2, CORO1B, PRPF4B, CHN2, CBX1, SLC26A2, TIMM50, ADORA2B, ADAMTS12, LAMA2, SF3A2, GATAD2A, FIRRE, ACSF3, FLOT2, USP24, PTPRU, ENTPD1, SEPTIN7, DYNLT1, ANXA10, SNX9, ARHGAP26, ZFR, TGM3, ODAM, PSMD11, HMGB2, PFKM, NUP214, WNT11, CALB1, UFD1, LONP2, SZT2, TCN2, BGN, RAPGEF3, MPRIP, CYGB, HSPE1, ESS2, PLCB2, MYO5A, SLC25A22, BIK, SLC9A3, CAPZA2, CREM, RAB11FIP2, IPO8, MYH6, HACD3, ELMO2, PSMD3, PEPD, DOCK5, NHP2, MZF1, ABCA4, KPNA6, EVC2, CYB5R3, ANXA7, SLC39A14, TMPRSS6, STOM, RPS27L, CLNS1A, MIR105-2, SNRPE, FBN2, SLC6A3, F10, DNAJB6, RXFP1, ARHGEF28, SPOCK1, EDIL3, ARHGAP24, PIH1D1, OGN, RHCG, ERP29, GAD2, SSR3,

SEC61B, TECR, SLC4A1, STOML2, RNU1-4, HMGN1, RIF1, UBE3B, PSPC1, DYNLT3, MEP1B, THOC6, SLC6A4, PSMB4, ADK, RNU1-1, SLC35A4, DOCK7, GNA12, SNRNP40, SUGP2, MIR4286, WLS, SLC20A1, IFIT1, RFC5, IFITM3, COQ8A, MBD3, PTPRN, ATOX1, FZD5, SCARB2, PRPH2, SLC46A1, NUDT21, PHF6, PTS, RNU1-3, HTR1A, CNBP, YY1AP1, TSHZ3, SRP19, FBXO3, HNMT, CRB3, DAZAP1, EXOC2, SIRT5, S100A13, PALS2, ANAPC7, SERPINF2, ABCC10, NUP160, SLC5A3, PPP1R18, PPP6R3, BYSL, DHRS4-AS1, CHD1, MYCBP2, PEAK1, CDC16, MT-ND2, USP15, SEC61A1, SURF6, LINC00265, PACSIN2, CDC42BPA, ARHGEF1, RAB31, RBM25, RNVU1-18, RNU1-2, DDAH1, PDIA4, CUL9, GLE1, ATP6V1A, AJAP1, DLX5, CPSF3, RAB11FIP1, MYOC, KCTD10, CRHR1, MRPL11, FUBP3, HAS3, SLCO1B1, ANK1, COPB2, GOPC, UGT1A6, SLC12A3, VPS16, POLR3A, MOV10, SRP54, FKBP8, ADRA1A, TGM4, RAB8A, LIPC, AKAP9, NR1I3, XRN2, ORM1, PREX1, MTX2, NT5C2, GID8, SAP18, TXNDC5, CPVL, EPB41L2, DPAGT1, ENAM, SLCO2A1, PPP1R10, APBB1, KCNIP2, TACR3, PIP5K1C, MAGI3, SHMT2, SRSF4, BDKRB1, MERTK, PRKCSH, AP2A1, PARD3B, RAB1B, MID1, ARHGAP32, CTSA, STT3B, ATG16L1, BAIAP2, RAB5C, PDE2A, PYCR2, WASF2, VAPA, SMC2, UGGT1, PRKAG1, ENPEP, NBPF11, HTR2C, TUBB4A, PCMT1, LSM4, CRB1, MIR876, C19orf48P, PSMD13, ARCN1, CASQ2, SLC15A2, TRIM5, CYRIB, CHRM2, EPX, UBN1, AP2M1, GP1BA, FAM98A, ADRA1B, STARD8, DEFB103B, SSBP1, KCNJ13, PDE6D, UCHL5, PPIH, AREL1, CPT1A, FDX1, TRIO, GINS2, RPGR, WDR1, NUP153, IGFL3, RAB21, RAB6A, FAM13A, PPM1G, COPG1, GPT2, CHMP2B, FIP1L1, SNTB2, OFCC1, UTP4, UCHL3, LTB4R, TUBD1, ALDOC, KPRP, SLC19A3, ARIH1, EBNA1BP2, VAPB, SMN1, KIF23, HTR1B, DDX42, IGBP1, RRS1, KCTD2, CLCNKB, SMC4, UBE3C, DMPK, ZNF346, TRMT112, BRPF1, KDF1, MIR374B, EXTL1, MAP7D1, ABCD3, EXOSC6, CDC42BPB, FMOD, APOC2, PDE6B, ATP5PO, XYLT1, HCRTR1, SNRPF, IL31, LAPTM5, MPZL1, INTS12, CPSF4, FAM20A, TMEM231, GAR1, WAPL, NMD3, ANOS1, TRN-GTT2-5, GUCY2D, KIF13B, IL11RA, TMPRSS4, SLC4A1AP, VAT1, INTS4, EVC, EPB41L1, CGN, MRPS23, EIF4G2, P2RY11, DDOST, PTPRM, SPOCK3, TBC1D4, TRPM4, MTPN, UBE2S, TRIM62, NPPC, TMX1, RSL24D1, MYCBP, ATAT1, TRN-GTT2-7, MESD, GPR18, SFXN1, UNC45A, CLDN16, ARL6IP5, RABEP1, DCAF8, PSMB1, ZMYM2, STX12, PREX2, C9orf78, NHS, SMNDC1, UBR1, DOCK2, MIR1260A, MEP1A, KPNA3, ODF2, KLKB1, HRH1, ATP8B1, RTKN, MAL, HTR2B, ARVCF, KCNJ2, MYH7, SGCB, SULT1A3, KIRREL3, ZNRD2, CALU, AHCYL1, CSTF1, DLX6, SORL1, ADAM28, ABCC9, TRN-GTT2-6, TRN-GTT2-1, TRN-GTT2-3, TRN-GTT2-4, MYO10, PDC, LTBP2, MNAT1, SLC40A1, CHN1, CLCN2, TRIR, CHST3, ADAM19, ARAP3, RNF114, RAVER1, HSPA4L, MEF2D, NKX2-8, KCNJ1, EMG1, PTRH1, FARSB, PGC, PPFIA3, DOCK4, TMEM41B, QPCTL, SNAP23, TCTN2,

GOLGA4, SMOC1, ITPA, POLDIP2, ACAT1, SRGAP2, COL10A1, URGCP, ABLIM1, ANKRD54, IL20, LHX6, CHAT, PIEZO1, CNPY1, ACSL1, TOMM40, VPS28, MLF2, CAPN3, GUCY1A1, SETX, CYP2C8, ERAL1, ZBTB33, ECI2, WDR82, TRIP11, SLC22A13, TRIM35, KCNE1, CEP89, OPN4, CSGALNACT1, KCND3, VAMP2, STX8, NHERF2, STRN3, CD52, NSUN5, MLPH, ARHGEF3, MYL6B, SLC39A3, PPIF, PATJ, SUPT6H, SYNJ2BP, RNF144A, HECTD1, DCTN2, SSR4, SLC7A1, SLC27A4, OPRD1, NCBP1, AQP9, P2RY12, GREM2, MIR133A2, FGD1, IL12RB2, DYNC1LI1, MIR576, PIGN, SUMO3, NRDC, ARHGEF26, NUP133, DUS3L, ARHGEF40, SRI, AP1M1, NUP205, THOC5, MRPS28, SLC25A24, ARHGAP11A, RBM22, SNORD7, DLD, BMP3, ACAA1, FBXO28, AGFG1, CPSF2, EHD1, SLC23A2, PARD6B, DOCK3, SCAMP3, MTM1, AJUBA, POLR3K, ATP5F1C, CCL24, WNT9B, ARHGAP23, STK38L, EPB41L4B, NEBL, SLC30A7, DOCK1, ARAP1, DCTN5, SLCO2B1, NTSR2, KLK1, ARHGAP21, EIF2B4, TFR2, NPC2, EIF2AK1, SF3B6, ZMPSTE24, DHX36, KREMEN1, PPIL4, APPAT, SLC2A9, ATP5MF, PRKX, NUP50, STT3A, NOC2L, TNRC6A, TOR1AIP1, DPY30, POU2AF2, DHPS, ASAP2, EML1, PLEKHG6, ARHGEF12, EFR3A, ARHGAP12, PRKRA, SPCS3, ELMO1, PDHA1, MPP3, ABCD1, SBSN, POLR3B, ATP5PD, SNX1, DYSF, MSBP1, CREBZF, DLGAP1, PLRG1, FARP2, SLC4A2, PPT1, RABEP2, ARHGEF39, CNNM4, MIA3, STRIP1, MUC20, ARHGEF25, RPRD1A, ACOX1, CLPTM1, CSRP2, VSIG4, BRIX1, CLCC1, SRBD1, FAM83H, GNAT1, SLC12A1, VDAC3, PFDN1, GPBAR1, JPH4, SCP2, NPR1, ATP5IF1, AHCYL2, RCL1, ARHGEF11, RAB22A, ZNF24, LTBP3, CHCHD3, NKD2, ADRA2A, SLC25A12, PPP6R2, SNX5, TPPP3, MIA-RAB4B, SIPA1L1, MYO15A, ZNF292, MTCH2, CRMP1, ELSPBP1, CHP1, SNRPG, PLPBP, CORIN, GNG2, PSME4, MFAP1, SLC2A8, LMLN, ASGR1, CIT, PCBD1, LSM12, PSMD7, NBPF8, PLEKHA7, SERTAD3, ARHGAP10, ARAP2, ARHGAP17, MAT2B, ARHGAP36, SLCO1A2, MRPS18B, SRSF8, HERC4, GJA9-MYCBP, MCCC1, RAB11B, FTCD, SPTBN2, AMT, CTTNBP2, ZC3H14, PPP2R5E, CETP, CHMP6, ICMT, ARHGEF18, HCCS, CLTA, MYLK3, FIS1, B4GALT7, SLC47A1, KCNJ12, SURF4, CCDC50, TRPC3, TACR2, CYRIA, ACTR1A, SLC16A2, NAALADL2, ASPM, NGB, MYBPC3, KCNE2, SNRPB2, ELK4, CEP162, BTBD7, NPHP3-ACAD11, HAUS5, ABRAXAS2, PGAP3, MGAT2, P2RX1, SGSH, PHRF1, LRRC41, MRPL4, PSMB6, CTSO, MRPL43, RHOG, SLCO6A1, CLEC18A, ERLIN1, ALDH18A1, NHLRC1, MLLT11, ANP32B, SLC10A2, CHPF, WDR74, KLF3, SLC29A4, EBI3, ECPAS, PNKD, SLC22A6, STK24, LSS, MC3R, SLC35A2, CSTF2, CD2BP2, BCKDHB, SLC26A3, VIL1, KRT84, CWC15, SLC10A1, NBPF9, RFX2, COPE, RPS19BP1, SEC11C, PDCD2, MT-ATP6, GYPC, ANKFY1, DOCK10, USP47, CACNB2, STRN4, POTEE, SLC5A6, ANKRD52, MYO9B, FARP1, CETN3, NOSIP, WBP11, PRCP, ARHGAP15, SMC5, DNMBP, SRPRA, NADSYN1, KIF3B, AP2A2, NSMCE2, NOA1, CRYGC, MARCHF8, CNGB3,

UBR2, SNAP29, EXOC5, RPGRIP1, NPTX1, RRP15, SLC4A3, WFDC12, KCMF1, FXYD2, MTCL1, SLC14A1, VAMP1, NBPF14, CCDC137, COL5A2, EHD3, KRTCAP2, SLC13A5, VMAC, ARHGAP20, NBPF20, MRPS31, USP16, NINL, SCAMP2, CTDSPL2, EXOC3, NBPF3, MARVELD2, RAPGEF2, GFM1, DOCK6, GRSF1, ZNF181, RNF169, FOXI3, LCT, IFT122, SRPRB, LTN1, ABR, PMP2, PSMB2, ITPR2, GABRB3, PTGFRN, ENSG00000277701, LOC100506076, CTF1, PAAF1, SLAIN2, POLR3H, SDF2L1, PFDN6, FGD2, NBPF15, MCMBP, MYO9A, PARD6G, NIPAL4, KRT222, HMGN5, ARMC8, GNE, FFAR2, PLEKHG3, CHRNA1, TXNDC12, TIAM2, SNX2, ARHGAP18, WDR59, NBPF10, MRPS35, ZNF768, ZNF207, DSEL, CLN5, CDC42BPG, KCNK3, DBNL, NAPA, CNGA3, NDUFA4, NBEA, CSGALNACT2, SMTNL2, ATP6V0E1, NPHP4, YME1L1, CKM, KIF17, MIR612, CLN3, GINS4, DAD1, NKD1, SLC7A9, CCDC86, POLR2D, PPIP5K2, NOXA1, RAB11FIP3, AMY2B, UBAC2, GDF6, PSMA2, KRT86, FGD5, ANGPTL7, SLC1A1, ZNF362, VILL, ANKRD28, TLR10, NAALADL1, MYOT, SLC26A9, SERPINA7, PJA2, SORBS2, ADGRG3, BSND, CELSR1, PDCD2L, LRCH3, FCHSD2, LAPTM4A, VAMP3, DNAJC11, CDCA5, WDR19, TRMT10C, PDE12, FAM76B, SLC3A1, SPSB1, UBL4A, STX7, ARHGAP6, DHRS7B, VPS33A, KRT81, MIR1249, TMOD2, DEFA5, TMOD1, MAP3K12, MED17, PLEKHG1, SLX9, HEATR1, CCDC85B, CNKSR2, WDTC1, EVL, TMEM147, LHB, VSX2, MRPS26, SELENOH, RAB34, SGCG, AMTN, NCAPD3, NDST1, ECH1, SNORD55, HBG2, PHPT1, BCKDHA, EXOC1, HBA2, MTMR14, CHST14, MGAM, NSRP1, RAB5B, STEEP1, ITSN2, CCDC12, AKIRIN2, SLC20A2, DLX1, HECW1, SUGT1, MRPL14, ACOT9, PHETA1, SPTB, GPRC5B, GLOD4, ARR3, MRPS17, PPIE, SEPHS1, WIPF2, THOC7, NUP42, MAP3K13, SLIRP, ITSN1, CACNA1B, CGNL1, VKORC1, ABCB7, ARHGAP22, ANKRD13A, PIK3R4, CYBRD1, CKMT1A, NDUFA5, KNOP1, GIT2, BARX1, ERP44, MRPS6, HECTD3, CRYBA4, MYOM2, SLC6A12, PLEKHG4B, ARHGEF19, SH3BP1, CRYBB2, CRPPA, PHKA1, PEX14, CLPX, SLC26A7, KAZN, ARHGAP8, SF3B5, RMND5A, CSPP1, TOMM22, NPHP3, FAM20B, SLC4A4, SLC23A1, DTWD1, WDR33, CHCHD2, EPS15L1, GJC1, PAN2, TFB1M, UCP3, HMGN4, EFR3B, RNF19A, SLC36A1, FYTTD1, DEPDC1B, PLN, SLC22A8, ZFC3H1, ZZEF1, BLOC1S2, GNAL, KIFAP3, AAMDC, AGAP3, CAMSAP3, CEP128, VAMP8, NPHP1, SLC35A3, REPIN1, MRPL10, NME1-NME2, SLC51A, TMPRSS13, REPS1, SLC22A12, DTNA, SAA4, ZNF22, MYT1, GGCX, NPR2, SLC15A3, MOB2, CRYBB3, MRPL16, HIGD1A, PRPF38A, DDX19B, LSM11, MARVELD3, SHPRH, BOLA2, KCNJ16, TMPRSS11E, LANCL1, LRCH1, ARL1, GHITM, HELZ, TIMM13, CDK16, DNM1, CFI, TTPA, B3GALT6, FBP2, LOC102723566, MICALL2, SRFBP1, MACO1, ZNF699, OPTC, MGAT1, SLC19A2, YPEL1, DZANK1, TMEM161A, LRP4, SLC6A1, MYADM, HERC1, CLN6, ARHGEF10, AMN, FGD6, MRPL53, MIR4497, SH3GL2, CTTNBP2NL, TPMT,

FKBP3, ZNF568, MCRIP1, AHSP, ARHGAP4, RIN2, TCAP, PAM16, CCDC120, NBPF2P, ITM2C, LMBR1, WDR83, CRYZL1, ARHGAP40, ZNF507, NOXO1, POLR3GL, EID3, HECW2, RDH5, B3GAT3, PEX6, SLC43A3, UQCC2, A1CF, SNAP47, HTR4, ZCCHC9, ENPP5, APMAP, INVS, CLDN17, RBM45, CPD, PDZK1, EXOC6, SLC14A2, ACR, FTSJ1, ARHGEF6, CCDC66, ARHGAP28, FGD4, LIPF, C5orf24, HMGN3, C12orf57, SIPA1L3, ROM1, FAM3B, OSR2, CKMT1B, TANC2, ARHGAP25, TMEM263, HTR1D, CCDC85C, POLRMT, KIFC3, STRIP2, ADRA2C, TMX2, GPR162, PLEKHG5, MCF2L2, SPCS1, ABCB8, TPRA1, B4GALT6, TAGAP, RPIA, AP1M2, TRIM41, BIN3, JPH1, NGEF, FMO1, CRB2, MOB4, B4GALNT2, MRPS14, COBL, SNORD1A, NDUFA8, CBLIF, CASD1, HRH3, CLPP, ABCG5, RAB6B, TTC8, ALS2, ARHGAP31, FAM13B, NOX3, WNK4, DOCK9, SNX18, SEC22B, SMC6, ARHGAP33, IL26, NCAPH2, P2RX4, RAB8B, UTP11, MCF2L, MCAT, NBPF19, ATP5ME, LRCH2, FGD3, GBGT1, KIF25, SH2D3A, ADAMDEC1, PSD, ATP13A2, SLC39A8, TTC9C, PKD2L1, CACNB1, SLC47A2, AIPL1, SCOC, RABAC1, CLDN15, GMIP, ACTN2, HCN2, PPP2R3B, LMAN2, TOX4, WFDC3, ARHGAP9, TRMT6, SLC4A11, MRPL47, SP6, HMX1, ARHGAP30, MUC3B, ARHGAP44, CEP19, VPS37C, LRCH4, RASGRF2, SCN2A, CTNS, B3GALNT1, NDUFV3, SEMG1, SULT1B1, EDF1, SEPT5-GP1BB, MCOLN1, IFNW1, SIKE1, CHST6, GNAO1, ADAMTS19, PIANP, RAB13, LOC110806262, CLUL1, SLC15A4, ARHGEF16, SLC6A5, SLC52A3, MIR3194, CCDC85A, RPUSD4, ADAMTS20, SBF1, ARHGAP19, SYPL1, CCNT2, ADRA2B, SLC44A4, RBM33, NBPF18P, TULP1, HACD1, UBE2J2, TNMD, RIMS3, PNLIP, FASTKD1, HERC6, MOXD1, EVI5L, PLEKHG7, ARHGEF9, ODAPH, RAB17, PLEKHG4, MYBPC1, TNNT1, MGAT4B, FXYD1, CPO, NBPF6, CHPF2, ARHGEF10L, NSMCE1, SYDE1, ZFTRAF1, PREPL, SNAPIN, SCN2B, SYNGR2, ARHGAP39, IFITM2, RAB11FIP5, MRPL2, PDZD11, CORO1A, ATP4B, SNORD105, SERP1, TMEM123, PDIA2, TMBIM4, CARD19, SLC24A3, MIR4665, MAP7D2, NBPF21P, WDR44, GRM7, SNF8, NPC1L1, SESTD1, NSMCE3, LHX8, RCCD1, CHST12, BCRP1, CNGA2, HRCT1, AKAP5, ARHGAP42, VAMP4, SLC13A4, RAB30, PRR36, NSMCE4A, DPP6, CNKSR3, SLF2, LILRB3, KRTAP9-1, ARHGAP27, RBSN, SNX31, NDUFB2, SLC51B, TMEM176B, TACO1, SLC5A10, RDH12, TMPRSS11D, IRAG2, MZT2B, KU881768, DOK4, MCOLN3, SLC25A31, DPM2, PWRN3, NBPF7P, GUCY1B1, SLC41A1, FKBP1B, FGFR1OP2, COL9A3, SLC26A6, PTRHD1, RMND1, SLCO4C1, RADIL, KCNK12, COL9A1, TIMM23, VAX1, SLC35B4, CDHR5, SLC22A11, PLLP, CAPS, ARSG, POT1-AS1, COL9A2, LDLRAP1, ZACN, SYDE2, RIMS4, LIN7A, MALL, SLC39A5, PDF, TAMALIN, ECT2L, SLC26A11, SLC26A5, SLC26A1, MIR1244-1, DOCK11, HDX, RHBG, APCS, DAOA-AS1, TRDN, KCNK13, TMEM134, CYSTM1, F11, BTBD8, NCAM2, TRIM10, NEURL2, GNAT2, ABCA9, CLPS, SPAG11B, TJP3, PIPSL, SPIN3, TRX-CAT1-

4, SLF1, FKBP2, CLN8, FXYD4, TRAPPC6A, RHAG, TRX-CAT1-8, MIR1244-2, MIR1244-4, CLDN19, ZDHHC22, KCNV2, ZNF8, VAMP5, MAN2B2, C2orf66, TRX-CAT1-2, TRX-CAT1-1, TRX-CAT1-3, TRX-CAT1-7, TRX-CAT1-6, ASB15, ABCG8, MICOS13, TRL-AAG2-1, PLPPR3, GARIN1B, SHISA8, SMIM1, ARHGAP11B, TMPRSS7, TRL-AAG2-2, TRL-AAG2-4, TRL-AAG2-3, WFDC9, ASIC5, KRTAP4-1
