# Supplementary material for: Matrine Targets BTF3 to Inhibit the Growth of Canine Mammary Tumor Cells
Source: Int J Mol Sci. 2023 Dec 30;25(1):540. doi: 10.3390/ijms25010540 (PMC10779273; doi:10.3390/ijms25010540)
Supplement: Supplementary file 1 [file ijms-25-00540-s001.zip › Supplementary File S2.pptx]

## Slide 1
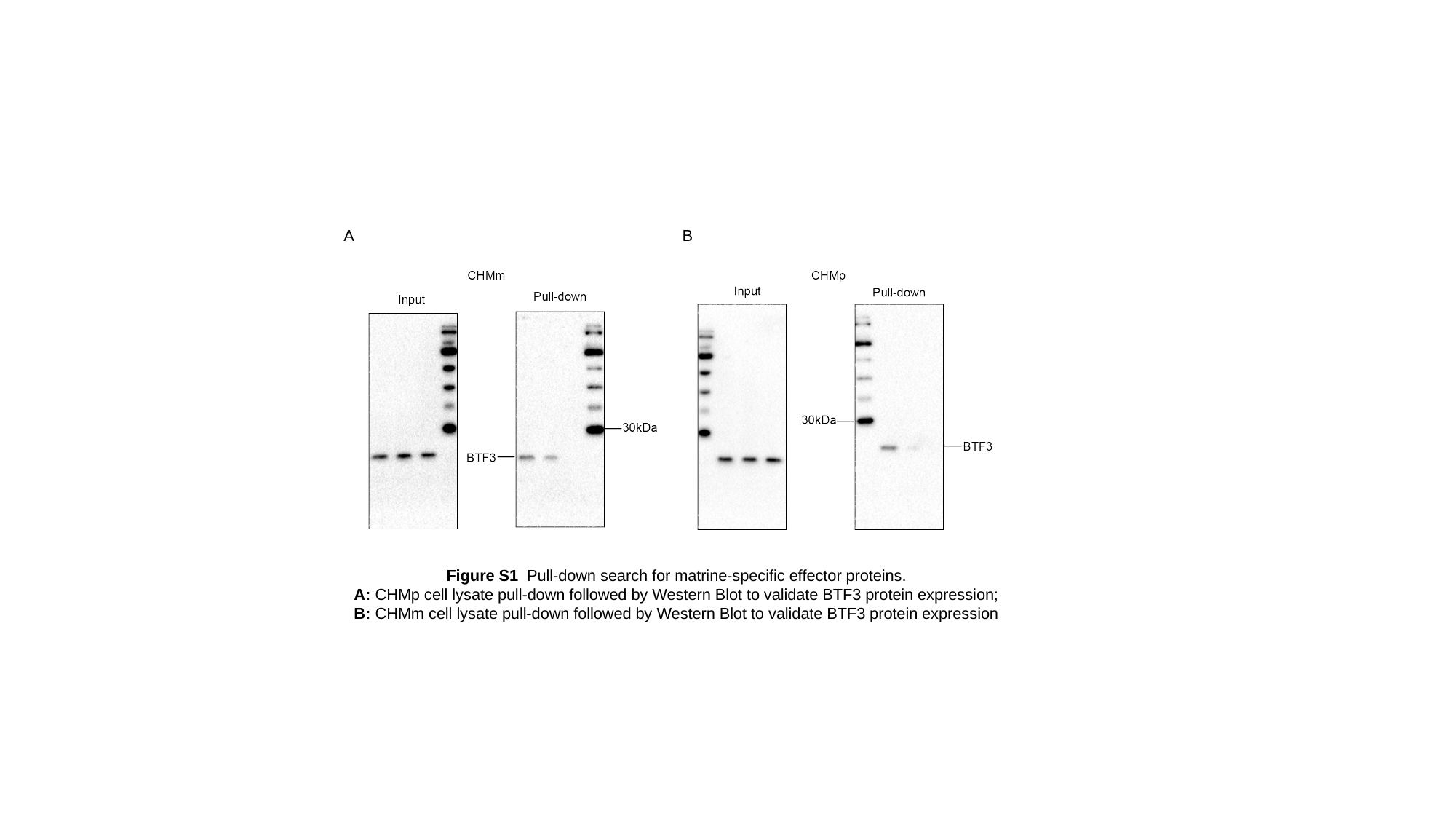

A
B
Figure S1 Pull-down search for matrine-specific effector proteins.
A: CHMp cell lysate pull-down followed by Western Blot to validate BTF3 protein expression; B: CHMm cell lysate pull-down followed by Western Blot to validate BTF3 protein expression

## Slide 2
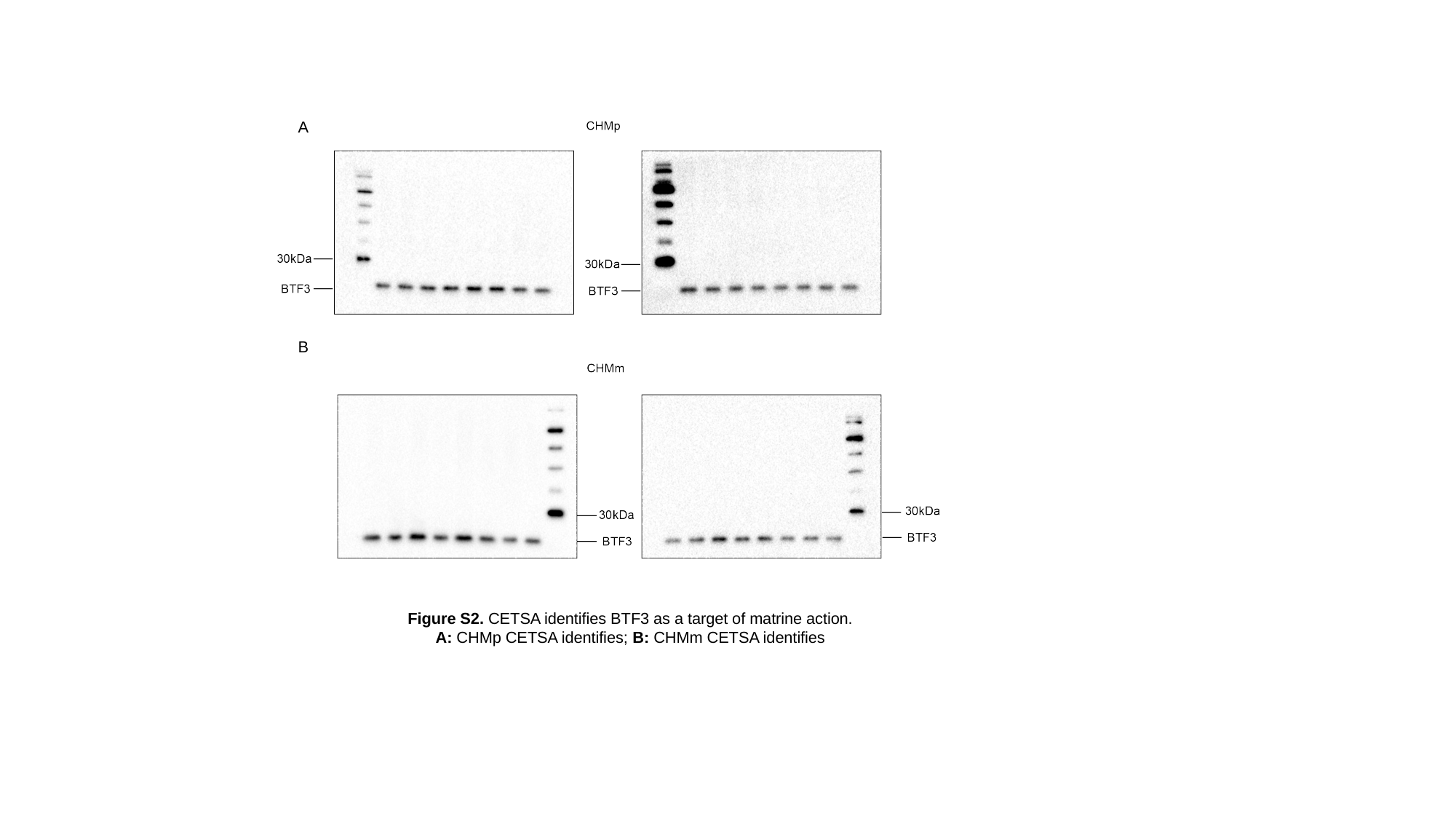

A
B
Figure S2. CETSA identifies BTF3 as a target of matrine action.
A: CHMp CETSA identifies; B: CHMm CETSA identifies

## Slide 3
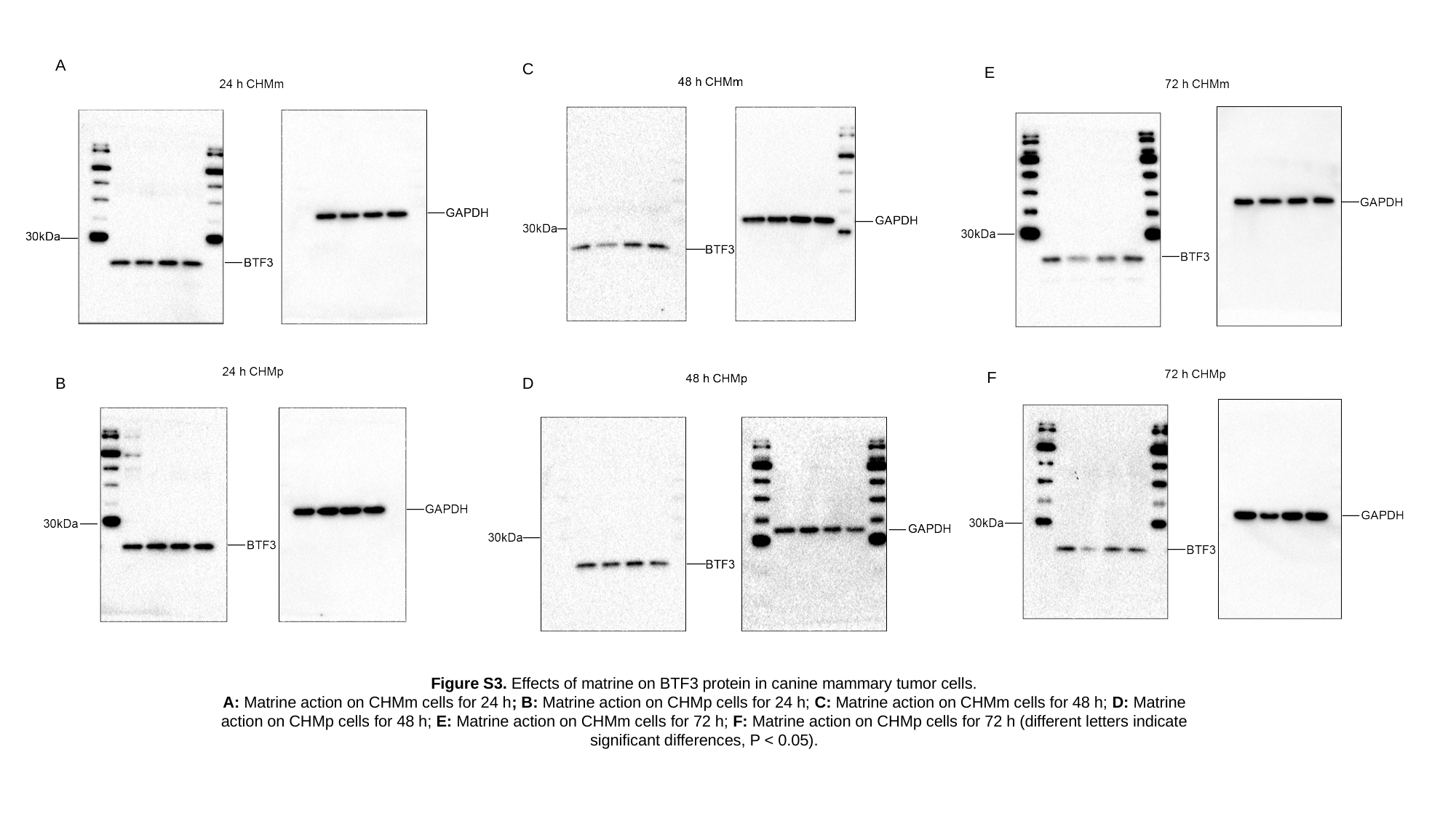

A
C
E
F
B
D
Figure S3. Effects of matrine on BTF3 protein in canine mammary tumor cells.
A: Matrine action on CHMm cells for 24 h; B: Matrine action on CHMp cells for 24 h; C: Matrine action on CHMm cells for 48 h; D: Matrine action on CHMp cells for 48 h; E: Matrine action on CHMm cells for 72 h; F: Matrine action on CHMp cells for 72 h (different letters indicate significant differences, P < 0.05).
